# YTHDF1 Is a Potential Pan-Cancer Biomarker for Prognosis and Immunotherapy

**DOI:** 10.3389/fonc.2021.607224

**Published:** 2021-05-06

**Authors:** Jian Hu, Dongxu Qiu, Anze Yu, Jiao Hu, Hao Deng, Huihuang Li, Zhenglin Yi, Jinbo Chen, Xiongbing Zu

**Affiliations:** Department of Urology, Xiangya Hospital, Central South University, Changsha, China

**Keywords:** YTHDF1, prognosis, tumor microenvironment, immune infiltration, immunotherapy, human cancer

## Abstract

**Background:**

YTH N6-methyladenosine RNA binding protein 1 (YTHDF1) has been indicated proven to participate in the cross-presentation of tumor antigens in dendritic cells and the cross-priming of CD8+ T cells. However, the role of YTHDF1 in prognosis and immunology in human cancers remains largely unknown.

**Methods:**

All original data were downloaded from TCGA and GEO databases and integrated *via* R 3.2.2. YTHDF1 expression was explored with the Oncomine, TIMER, GEPIA, and BioGPS databases. The effect of YTHDF1 on prognosis was analyzed *via* GEPIA, Kaplan-Meier plotter, and the PrognoScan database. The TISIDB database was used to determine YTHDF1 expression in different immune and molecular subtypes of human cancers. The correlations between YTHDF1 expression and immune checkpoints (ICP), tumor mutational burden (TMB), microsatellite instability (MSI), and neoantigens in human cancers were analyzed *via* the SangerBox database. The relationships between YTHDF1 expression and tumor-infiltrated immune cells were analyzed *via* the TIMER and GEPIA databases. The relationships between YTHDF1 and marker genes of tumor-infiltrated immune cells in urogenital cancers were analyzed for confirmation. The genomic alterations of YTHDF1 were investigated with the c-BioPortal database. The differential expression of YTHDF1 in urogenital cancers with different clinical characteristics was analyzed with the UALCAN database. YTHDF1 coexpression networks were studied by the LinkedOmics database.

**Results:**

In general, YTHDF1 expression was higher in tumors than in paired normal tissue in human cancers. YTHDF1 expression had strong relationships with prognosis, ICP, TMB, MSI, and neoantigens. YTHDF1 plays an essential role in the tumor microenvironment (TME) and participates in immune regulation. Furthermore, significant strong correlations between YTHDF1 expression and tumor immune-infiltrated cells (TILs) existed in human cancers, and marker genes of TILs were significantly related to YTHDF expression in urogenital cancers. TYHDF1 coexpression networks mostly participated in the regulation of immune response and antigen processing and presentation.

**Conclusion:**

YTHDF1 may serve as a potential prognostic and immunological pan-cancer biomarker. Moreover, YTHDF1 could be a novel target for tumor immunotherapy.

## Introduction

N6-methyladenine (m6A), occurring in the N6-position of adenosine, is the most abundant modification of mRNA and noncoding RNAs in mammals (1–3). Moreover, it plays an essential role in RNA nucleation, RNA-protein interaction, mRNA metabolism, mRNA stability and splicing, and mRNA translation (4, 5). Under normal conditions, m6A molecules modify the processing of RNA by serving as “writers”, “erasers”, and “readers” (6, 7). However, abnormal expression of m6A molecules has been proven to promote tumorigenesis by affecting oncogene expression (8–10). Recent evidence reveals that m6A produces a marked effect on cancer development, such as proliferation, migration, and invasion (11), which means that m6A molecules may be promising prognostic biomarkers and therapeutic targets.

YTH N6-methyladenosine RNA binding protein 1 (YTHDF1) belongs to the YTH domain family, and acts as a “reader” of m6A-modified mRNAs. In the cytosol, YTHDF1 plays an essential role in RNA translation initiation by binding to initiation factors (4). Previous studies proved that tumor neoantigens could cause spontaneous antitumor immune responses and predict the response to cancer immunotherapies (12, 13). Recent studies have demonstrated that YTHDF1 plays a crucial role in cancer development and durable neoantigen-specific antitumor immunity (4). For example, YTHDF1 promotes the progression of lung cancer by participating in the m6A demethylase the ALKBH5 pathway (14). In colorectal cancer, YTHDF1 is regarded as an oncogene (15); In breast cancer, overexpression of YTHDF1 predicts worse survival (16). Furthermore, YTHDF1 inhibits the function of antitumor immune cells in the tumor microenvironment (TME) by acting on the infiltrated level of CD8+ T cells and natural killer cells. Blocking the YTHDF1 reactivates the inhibited antitumor immunity and synergistically improves the therapeutic efficacy of anti-PD-L1 inhibitors (17). All of the above studies indicate that YTHDF1 might be a promising prognostic and therapeutic pan-cancer biomarker.

However, the role of YTHDF1 in prognosis and immunology in human cancers has seldomly been analyzed systematically. In this study, the role of YTHDF1 in prognosis and immunology in human cancers was comprehensively analyzed. Furthermore, the potential association between YTHDF1 expression and immune subtypes, molecular subtypes of different cancer types, promising immune biomarkers and tumor-infiltrating lymphocytes (TILs) in the TME was analyzed. In addition, we explored the effect of YTHDF1 expression in urogenital cancers to verify the results in human cancers. This study aimed to explore the potential of YTHDF1 in anticancer immunotherapy in human cancer, thus offering insight to a new antitumor strategy.

## Methods

### Data and Software Availability

All original data were downloaded from The Cancer Genome Atlas (TCGA) (https://cancergenome.nih.gov/) and Gene Expression Omnibus (GEO) (https://www.ncbi.nlm.nih.gov/geo/) databases. Detailed information on human cancers and corresponding individual sample sizes is displayed in **Supplementary Table 1**. R 3.2.3 was used to integrate the original data and verify the results analyzed by the website database. All applied online web tools were introduced below.

### Four Databases to Analyze YTHDF1 Expression in Human Cancers

The Oncomine database (https://www.oncomine.org/resource/login.html), TIMER database (https://cistrome.shinyapps.io/timer/), and GEPIA database (http://gepia2.cancer-pku.cn/#analysis) were used to compare YTHDF1 expression between human cancers and paired normal tissue (18–20). The BioGPS database (http://biogps.org) was used to analyze the expression profiles of YTHDF1 in different cancer and paired normal cell lines (21). In the Oncomine database, we set the thresholds to 0.001 for P-value and 1.5 for the fold change.

### Three Databases to Analyze the Prognostic Value of YTHDF1 in Human Cancers

The GEPIA database (http://gepia2.cancer-pku.cn/#analysis), Kaplan-Meier Plotter database (http://kmplot.com/analysis/), and PrognoScan databases (http://dna00.bio.kyutech.ac.jp/PrognoScan/index.html) were used to explore the prognostic value of YTHDF1 expression in human cancers (20, 22, 23). The GEPIA database is an online website, and its analyzed tumor and normal tissue data were from the TCGA database. We used the GEPIA database to explore the correlation between YTHDF1 expression and overall survival (OS) and disease-free survival (DFS) in 33 cancer types. In the GEPIA database, the median YTHDF1 expression was used as a cutoff value to classify groups. The Kaplan-Meier Plotter database classifies groups by calculating an optimal cutoff value automatically. By using the Kaplan-Meier Plotter database, we identified the association between YTHDF1 expression and OS and relapse-free survival (RFS) in 21 cancer types. Hazard ratios (HRs) with corresponding 95% confidence intervals (CIs) and log-rank P-values were calculated. Data on the PrognoScan website were also collected from the TCGA and GEO database. The prognostic value was considered statistically significant when the P-value was less than 0.05.

### Database Applied to Analyze YTHDF1 Expression in Immune and Molecular Subtypes of Human Cancers

The TISIDB database (http://cis.hku.hk/TISIDB/index.php) is an online integrated repository portal collecting abundant human cancer datasets from the TCGA database (24). The correlations between YTHDF1 expression and immune or molecular subtypes of different cancer types were explored *via* the TISIDB database. Differences with a P-value < 0.05 were considered to be statistically significant.

### Database Used to Analyze the Correlation Between YTHDF1 Expression and Immune Checkpoint (ICP) Genes, Tumor Mutational Burden (TMB), Microsatellite Instability (MSI), Neoantigen, and ESTIMATE of the TME in Human Cancers

The relationship between YTHDF1 expression and immune checkpoint (ICP) genes, biomarkers and ESTIMATE score in the TME was explored *via* the SangerBox website (http://sangerbox.com/Tool), which is a useful online platform for TCGA data analysis (25). Immune checkpoint (ICP) genes were selected according to a previous study (26). Tumor mutational burden (TMB), microsatellite instability (MSI), and neoantigens have been proved to be important biomarkers of the TME (27–29). ESTIMATE (Estimation of Stromal and Immune cells in Malignant Tumor tissues using Expression data) was an algorithm designed by Yoshihara et al. for predicting tumor purity in TME, and this algorithm included stromal score (that captures the presence of stroma 136 in tumor tissue), immune score (that represents the infiltration of immune cells in tumor 137 tissue), and estimate score (that infers tumor purity) (30). Correlations between YTHDF1 expression and the above TME biomarkers were explored *via* the SangerBox website. Differences with a P-value < 0.05 were considered to be statistically significant.

### Three Databases Used to Analyze the Correlation Between YTHDF1 Expression and Immune Infiltration Cells and Their Marker Genes

We first explored the correlation between YTHDF1 expression and six immune cells (B cells, CD4+ T cells, CD8+ T cells, neutrophils, macrophages, and dendritic cells) in the TME of 31 human cancers *via* the SangerBox website. Then, the correlation between YTHDF1 expression and ten immune cells in the TME was explored in six urogenital cancers by the SangerBox website. These ten immune cells included B cells lineage, CB8+ T cells, cytotoxic lymphocytes, endothelial cells, fibroblasts, monocytic cells, myeloid dendritic cells, neutrophils, natural killer cells, and T cells. We reanalyzed the correlation between YTHDF1 expression and tumor infiltrating lymphocytes (TILs) *via* the TIMER and GEPIA databases to verify the above results. The TIMER database (https://cistrome.shinyapps.io/timer/), which collected 10897 samples across 32 cancer types from TCGA, was created to analyze the level of tumor-associated immune cell infiltration in the TME (19, 31). Then, we verified the correlation between YTHDF1 expression and 24 tumor-infiltrating lymphocytes (TILs) in six urogenital cancers *via* the GEPIA database (20). These 24 tumor-infiltrating lymphocytes (TILs) were selected from a previous study (32), and detailed information is displayed in **Supplementary Table 2**. Finally, the TIMER database was used to explore the relationship between YTHDF1 expression and marker genes of the main immune cells, including B cells, CD8+ T cells, follicular helper T cells (Tfh), T-helper 1 (Th1) cells, T-helper 2 (Th2) cells, T-helper 9 (Th9) cells, T-helper 17 (Th17) cells, T-helper 22 (Th22) cells, Tregs cells, exhausted T cells, M1 macrophages, M2 macrophages, tumor-associated macrophages, monocytes, natural killer (NK) cells, neutrophils and dendritic cells (33–35). The difference at a P-value < 0.05 was considered to be statistically significant.

### Database Used to Explore YTHDF1 Genomic Alterations in Six Urogenital Cancers

cBio Cancer Genomics Portal (c-BioPortal) (http://cbioportal.org) collected a multidimensional cancer genomics data set (36). Therefore, the c-BioPortal database was applied to explore YTHDF1 genomic alterations in six urogenital cancers.

### Database Used to Explore YTHDF1 Expression in Different Clinical Subgroups of Six Urogenital Cancers

The UALCAN database (http://ualcan.path.uab.edu) collected RNA-seq and clinical data of 31 cancer types from TCGA (37), and it offered a useful platform to analyze gene expression in tumor and normal tissues. This database was used to analyze the relationship between individual gene expression and clinicopathological features in human cancers.

### Database Used to Explore YTHDF1 Coexpression Networks

The LinkedOmics database (http://www.linkedomics.org/login.php) is a visual platform and is used to explore the gene expression profile (38). We used LinkedOmics to determine the YTHDF1 coexpression genes by using Pearson’s correlation coefficient and showed the results *via* heat maps, and volcano plots. Then, we explored the Gene Ontology biological process (GO_BP), and KEGG pathways of YTHDF1 and its coexpression genes by using gene set enrichment analysis (GSEA).

## Results

### YTHDF1 Is Significantly Differentially Expressed Between Tumors and Normal Tissues in Human Cancers

**Figure 1:**
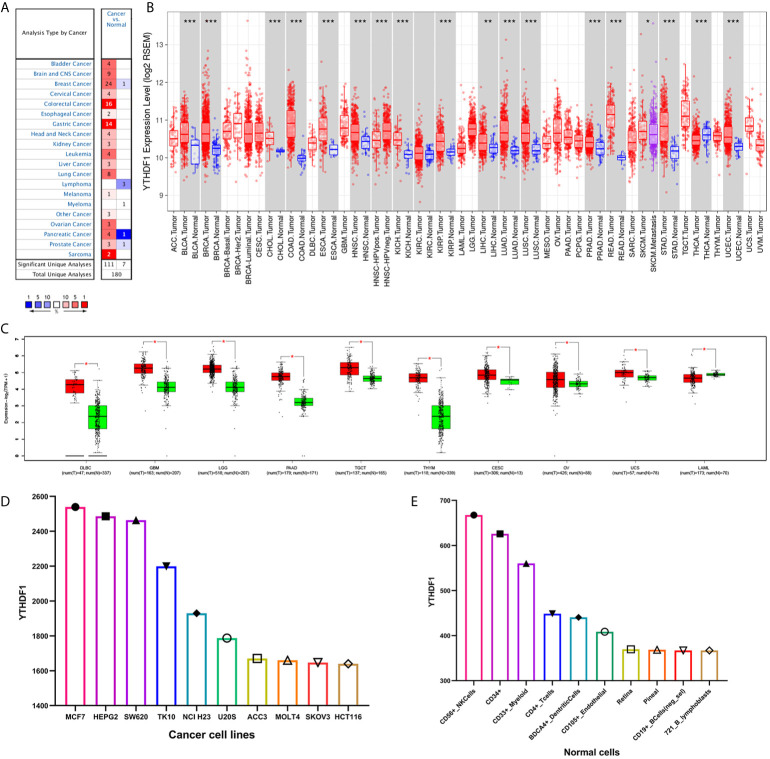
YTHDF1 expression levels in human cancers. **(A)** YTHDF1 expression in different cancers and paired normal tissue in the Oncomine database. **(B)** YTHDF1 expression levels in different cancer types from the TCGA database analyzed by the TIMER database. (*P < 0.05, **P < 0.01, ***P < 0.001). **(C)** YTHDF1 expression in several cancers and paired normal tissue in the GEPIA database. **(D)** the expression of YTHDF1 in different cancer cell lines analyzed by the BioGPS database. **(E)** the expression of YTHDF1 in normal tissue analyzed by the BioGPS database.

The Oncomine database showed that YTHDF1 mRNA levels were significantly higher in most human cancers, such as bladder, brain and CNS, breast, cervical, colorectal, esophageal, head and neck, kidney, liver, lung, ovarian, pancreatic, prostate cancer, leukemia, melanoma, sarcoma, and other cancer types compared to the corresponding normal tissues. In contrast, YTHDF1 expression was significantly lower only in lymphoma (**Figure 1A**). The results from the TIMER database showed that YTHDF1 expression was significantly higher in BLCA (bladder urothelial carcinoma), BRCA (breast invasive carcinoma), CHOL(cholangiocarcinoma), COAD (colon adenocarcinoma), ESCA(esophageal carcinoma), HNSC (head and neck cancer), KICH (kidney chromophobe), KIRP (kidney renal papillary carcinoma), LIHC (liver hepatocellular carcinoma), LUAD (lung adenocarcinoma), LUSC (lung squamous cell carcinoma), PRAD (prostate adenocarcinoma), STAD (stomach adenocarcinoma) and UCEC (uterine corpus endometrial carcinoma) than in adjacent normal tissue. However, YTHDF1 mRNA expression was low only in THCA (thyroid carcinoma) (**Figure 1B**). The results of the GEPIA database analysis are displayed as supplementary results of cancers without paired normal tissues in the TIMER database. Furthermore, the results showed that YTHDF1 mRNA expression was significantly high among most cancer types except LAML (acute myeloid leukemia), which was consistent with the Oncomine database results (**Figure 1C**). The expression of YTHDF1 in different cancer cell lines and normal tissues was investigated *via* the BioGPS database, and we found that YTHDF1 had a high expression level in almost all cancer cell lines. Ten cancer cell lines with the highest YTHDF1 expression level are displayed in **Figure 1D**. In normal cells, the YTHDF1 expression level in immune cells was the highest (**Figure 1E**). Detailed information is shown in **Supplementary Figure 1**. The above results suggested that YTHDF1 was overexpressed in cancer tissues and might participate in the process of immune regulation.

### YTHDF1 Is a Prognostic Pan-Cancer Biomarker

**Figure 2:**
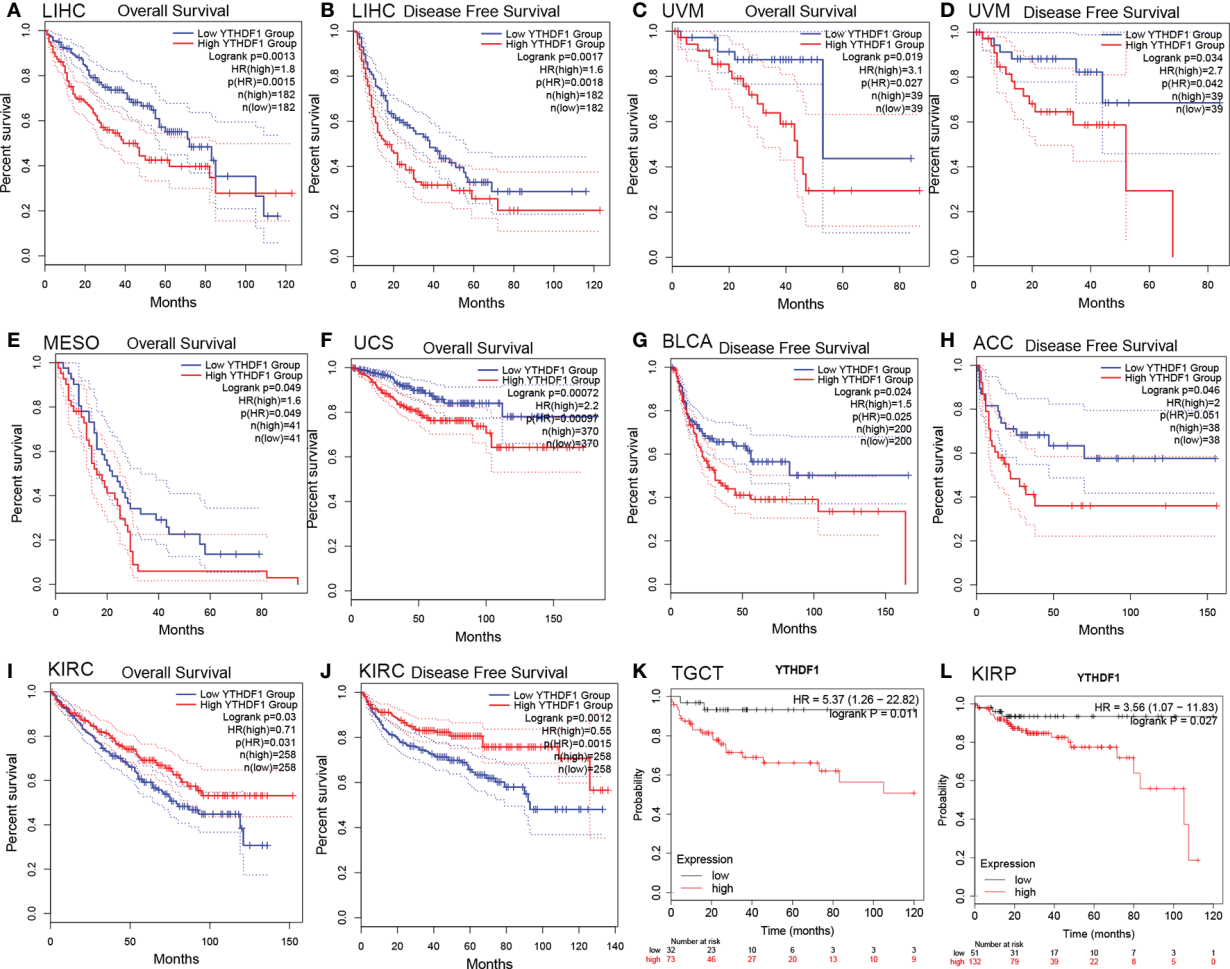
Kaplan-Meier survival curve of human cancers with high and low YTHDF1 expression analyzed by the GEPAI database **(A–J)** and the Kaplan-Meier plotter database **(K, L)**. **(A–D)** High YTHDF1 expression was related to worse OS and DFS in LIHC (n = 364) and UVM cohorts (n = 78). **(E, F)** High YTHDF1 expression was related to worse OS in MESO (n = 82) and UCS cohorts (n = 740). **(G, H)** High YTHDF1 expression was related to worse DFS in BLCA (n = 400) and ACC cohorts (n = 76). **(I, J)** High YTHDF1 expression was related to better OS and DFS in KIRC cohorts (n = 316). **(K, L)** higher YTHDF1 expression was related to poorer RFS in TGCT (n = 105) and KIRP (n = 183). OS, overall survival; DFS, disease free survival; RFS, relapse-free survival.

The prognostic value of YTHDF1 expression in human cancers was analyzed by several databases. In GEPIA, we found that higher YHTDF1 expression was associated with poorer overall survival (OS) and disease-free survival (DFS) in LIHC (n = 364, OS: HR = 1.8, P = 0.0015; n = 364, DFS: HR = 1.6, P = 0.0018; **Figures 2A, B**), and UVM (uveal melanoma) (n = 78, OS: HR = 3.1, P = 0.027; n = 78, DFS: HR = 2.7, P = 0.034; **Figures 2C, D**). In addition, patients with higher YTHDF1 expression had poorer OS in mesothelioma (MESO) (n = 82, HR = 1.6, P = 0.049; **Figure 2E**) and uterine carcinosarcoma (UCS) (n = 740, HR = 2.2, P = 0.00097; **Figure 2F**), and also had poor DFS in BLCA (n = 400, HR = 1.5, P = 0.025; **Figure 2G**) and adrenocortical carcinoma (ACC) (n = 76, HR = 2, P = 0.046; **Figure 2H**). However, higher YTHDF1 expression was related to better OS and DFS in KIRC (kidney renal papillary cell carcinoma) (n = 516, OS: HR = 0.71, P = 0.031; n = 516, DFS: HR = 0.55, P = 0.0015; **Figures 2I, J**). In the Kaplan-Meier plotter database, higher YTHDF1 expression was associated with poorer RFS in TGCT (testicular germ cell tumors) (n = 105, HR = 5.37, P = 0.011; **Figure 2K**) and KIRP (n = 183, HR = 3.56, P = 0.027; **Figure 2L**). More details of the relationship between YTHDF1 and OS or relapse-free survival (RFS) analyzed by the Kaplan-Meier plotter database are shown in **Supplementary Figure 2**. Furthermore, the correlation between YTHDF1 expression and survival in GEO datasets was evaluated by PrognoScan. Higher expression of YTHDF1 showed worse survival in breast, soft tissue, bladder, lung and brain cancer. Details are shown in **Supplementary Table 3**. The above results proved that YTHDF1 expression closely related to the prognosis of various cancer types.

### YTHDF1 Expression Is Related to Immune and Molecular Subtypes in Human Cancers

**Figure 3:**
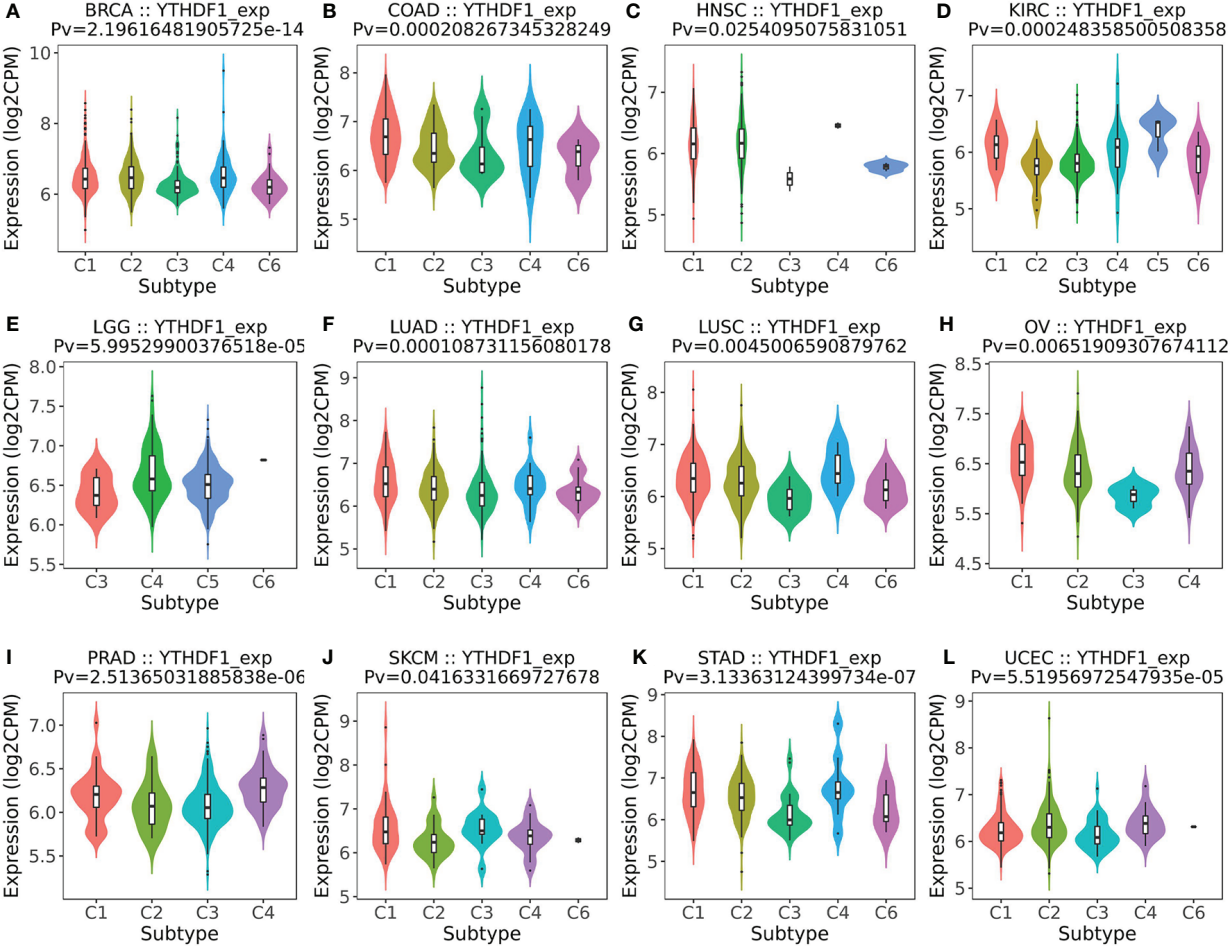
The relationship between YTHDF1 expression and pan-cancer immune subtypes. **(A)** in BRCA, **(B)** in COAD, **(C)** in HNSC, **(D)** in KIRC, **(E)** in LGG, **(F)** in LUAD, **(G)** in LUSC, **(H)** in OV, **(I)** in PRAD, **(J)** in SKCM, **(K)** in STAD, **(L)** in UCEC.

**Figure 4:**
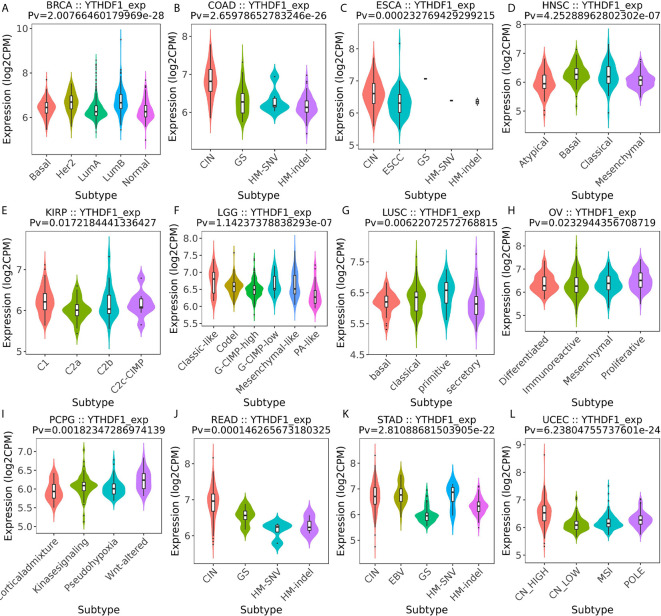
The relationship between YTHDF1 expression and pan-cancer molecular subtypes. **(A)** in BRCA, **(B)** in COAD, **(C)** in ESCA, **(D)** in HNSC, **(E)** in KIRP, **(F)** in LGG, **(G)** in LUSC, **(H)** in OV, **(I)** in PCPG, **(J)** in READ, **(K)** in STAD, **(L)** in UCEC.

Next, the role of YTHDF1 expression on immune and molecular subtypes among human cancers was explored with the TISIDB website. Immune subtypes were classified into six types, including C1 (wound healing), C2 (IFN-gamma dominant), C3 (inflammatory), C4 (lymphocyte depleted), C5 (immunologically quiet) and C6 (TGF-b dominant). The results showed that YTHDF1 expression was related to different immune subtypes in BRCA, COAD, HNSC, KIRC, LGG (brain lower grade glioma), LUAD, LUSC, OV (ovarian serous cystadenocarcinoma), PRAD, SKCM (skin cutaneous melanoma), STAD, and UCEC (**Figure 3**). Additionally, YTHDF1 expression differed in different immune subtypes of one cancer type. Taking KIRC as an example, YTHDF1 showed high expression in C1 and C5 types and low expression in C2 types. For different molecular subtypes of cancers, a significant connection with YTHDF1 expression existed in BRCA, COAD, ESCA, HNSC, KIRP, LGG, LUSC, OV, PCPG (Pheochromocytoma and Paraganglioma), READ (rectum adenocarcinoma), STAD and UCEC (**Figure 4**). YTHDF1 expression in different immune and molecular subtypes of other cancers is shown in **Supplementary Figures 3**, **4**. Based on the above results, we concluded that YTHDF1 expression differs in immune subtypes and molecular subtypes of various human cancer types.

### YTHDF1 Expression Is Related to Immune Checkpoint (ICP) Genes in Human Cancers

**Figure 5:**
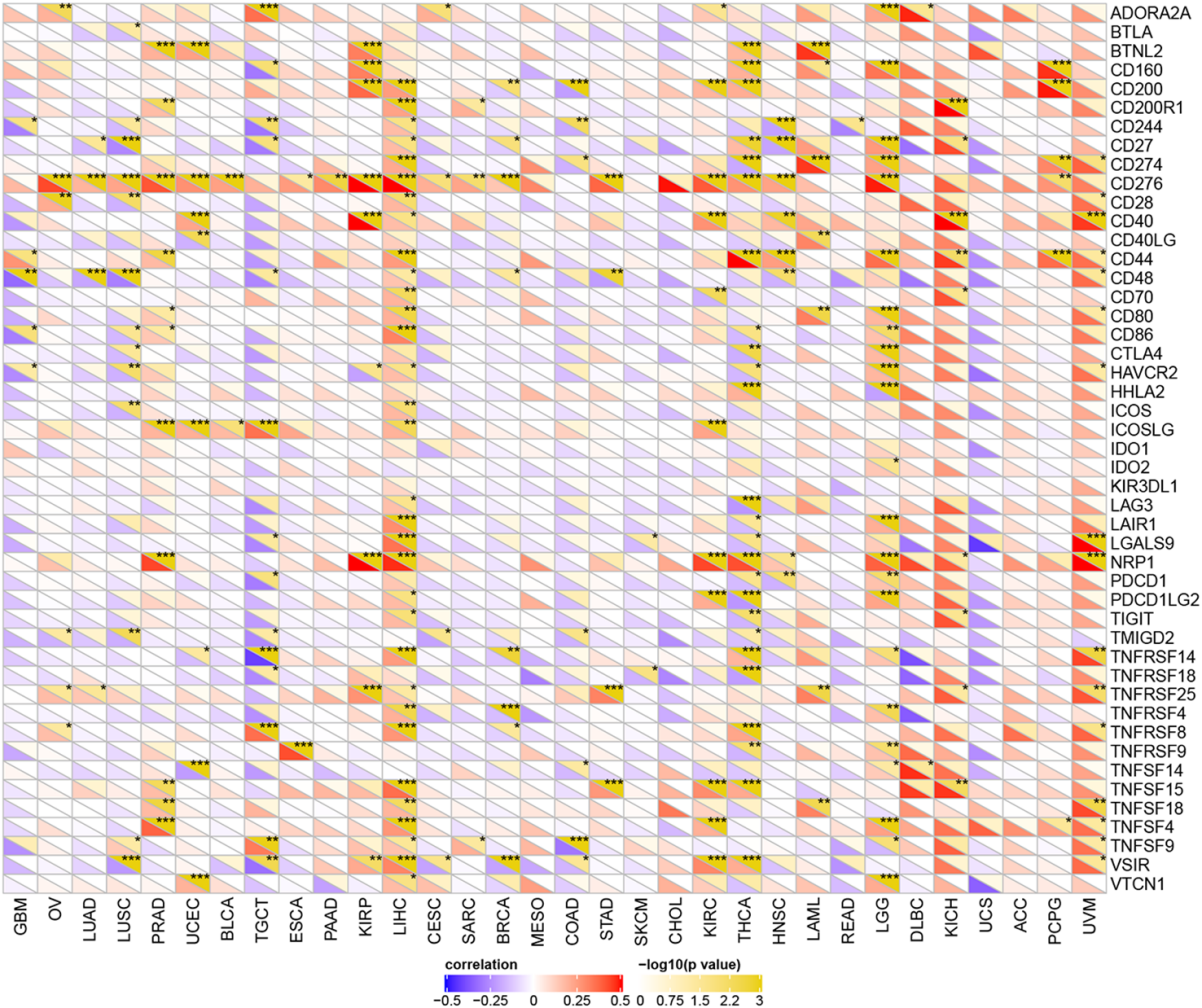
The relationship between YTHDF1 expression and pan-cancer immune checkpoint genes. *P < 0.05; **P < 0.01; ***P < 0.001.

Studies have proven that immune checkpoint (ICP) genes have a great influence on immune cell infiltration and immunotherapy (39). Subsequently, we explored the associations between YTHDF1 expression and ICP genes in human cancers to explore the potential of YTHDF1 in immunotherapy. Among 47 ICP genes, strong relationships with YTHDF1 expression were found in many types of cancer, such as GBM (glioblastoma multiforme), OV, LUSC, PRAD, UCEC, TGCT, KIRP, LIHC, BRCA, COAD, KIRC, THCA, LAML, LGG, KICH, and UVM (**Figure 5**). In OV, PRAD, UCEC, KIRP, LIHC, KIRC, LAML, LGG, KICH, and UVM, YTHDF1 expression was positively related to immune checkpoint genes, especially in LIHC, in which 32 of 47 immune checkpoint genes had connections with YTHDF1 expression. This suggests that YTHDF1 might coordinate the activity of these ICP genes in different signal transduction pathways, and potentially serve as an ideal immunotherapy target. And high YTHDF1 expression might predict good therapeutic efficacy of immunotherapy targeting ICP genes. In GBM, LUSC, TGCT, BRCA, COAD and THCA, YTHDF1 is negatively related with ICP genes, which means that high YTHDF1 expression might predict unsatisfying immunotherapy outcomes when targeting ICP genes. On the other hand, YTHDF1 inhibitors might be potential alternative treatments. Thus, we hypothesized that YTHDF1, working as a potential pan-cancer biomarker or as a new immunotherapy target, may predict the immunotherapy response or achieve a promising therapeutic outcome, respectively.

### YTHDF1 Expression Is Related to Tumor Mutational Burden (TMB), Microsatellite Instability (MSI), Neoantigen, and ESTIMATE

**Figure 6:**
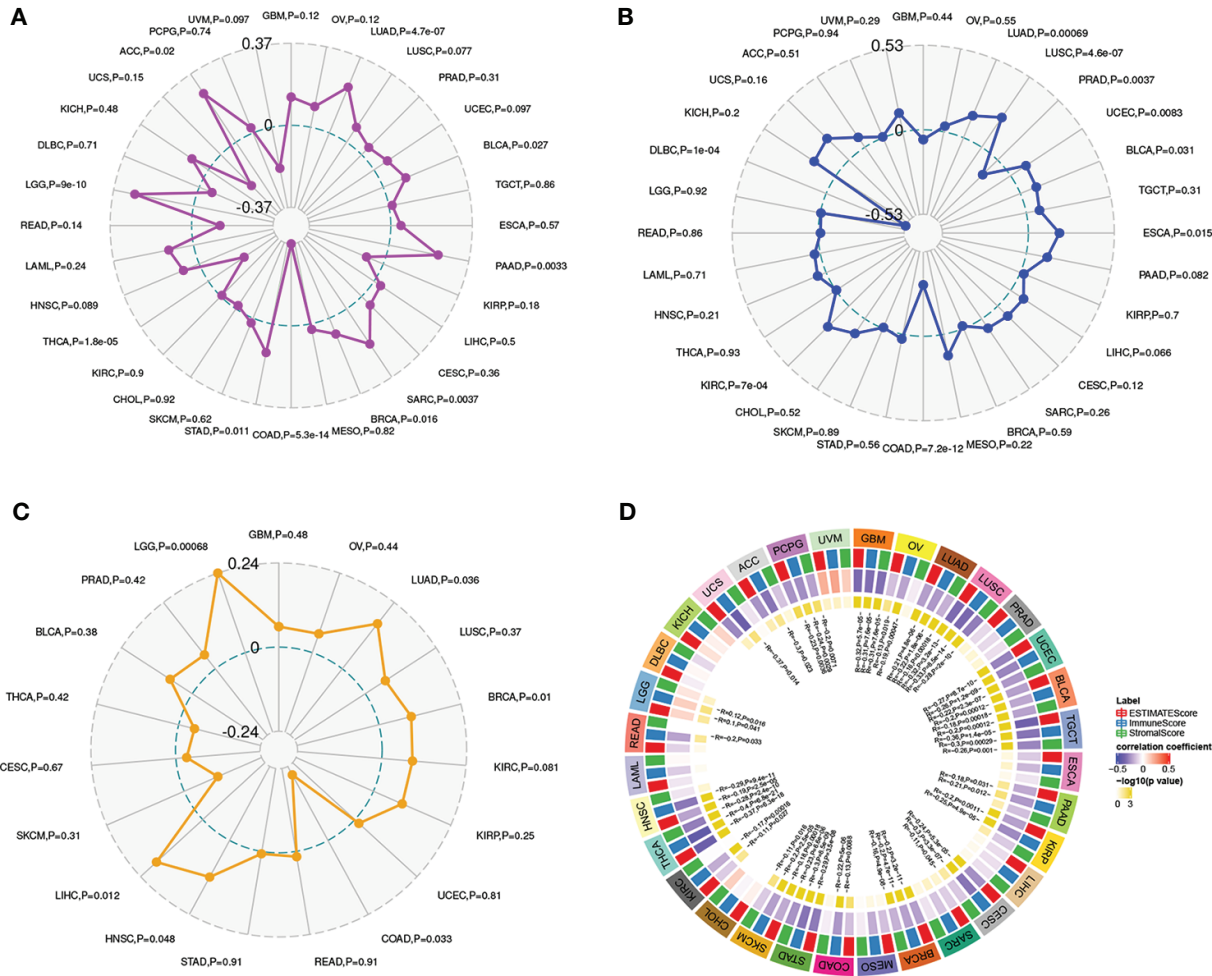
The relationship between YTHDF1 expression and TMB **(A)**, MSI **(B)**, neoantigen **(C)** and ESTIMATE score **(D)** in human cancers. TMB, tumor mutational burden; MSI, microsatellite instability; MMR genes, mismatch repair genes; ESTIMATE, Estimation of Stromal and Immune cells in Malignant Tumor tissues using Expression data.

To explore the role of YTHDF1 in the immune mechanism and immune response of the tumor microenvironment (TME), we analyzed the correlations between YTHDF1 expression and TMB, MSI, and neoantigens. TMB, MSI, and neoantigens in tumor microenvironment are related to antitumor immunity and could predict the therapeutic efficacy of tumor immunotherapy (13, 40–42). Our results showed that YTHDF1 expression had significant positive associations with TMB in LUAD, LUSC, BLCA, PAAD (pancreatic adenocarcinoma), SARC (sarcoma), BRCA, STAD, LGG, and ACC and negative relations in COAD and THCA (**Figure 6A**). For MSI, there were positive correlations with YTHDF1 expression in LUAD, LUSC, UCEC, BLCA, ESCA, and KIRC and negative correlations with in PRAD, COAD, and DLBC (lymphoid neoplasm diffuse large B-cell lymphoma) (**Figure 6B**). Analyzing the relationships between YTHDF1 expression and neoantigens, we found that YTHDF1 expression was positively related to neoantigens in LUAD, BRCA, HNSC, LIHC, and LGG and negatively related to neoantigens in COAD (**Figure 6C**). Then, we explored the associations between YTHDF1 expression and three kinds of ESTIMATE (**Figure 6D**). The results indicated that there were strong positive correlations with YTHDF1 expression in LGG and negative correlations in ACC, PCPG, GBM, OV, LUAD, LUSC, UCEC, BLCA, TGCT, PAAD, KIRP, CESC, BRCA, COAD, STAD, SKCM, KIRC, THCA, HNSC, READ, and UCS. The above results further verified our conjecture that YTHDF1 may influence antitumor immunity by regulating the composition and immune mechanism in the TME.

### YTHDF1 Correlates With Immune Cell Infiltration in the TME in Human Cancers

**Figure 7:**
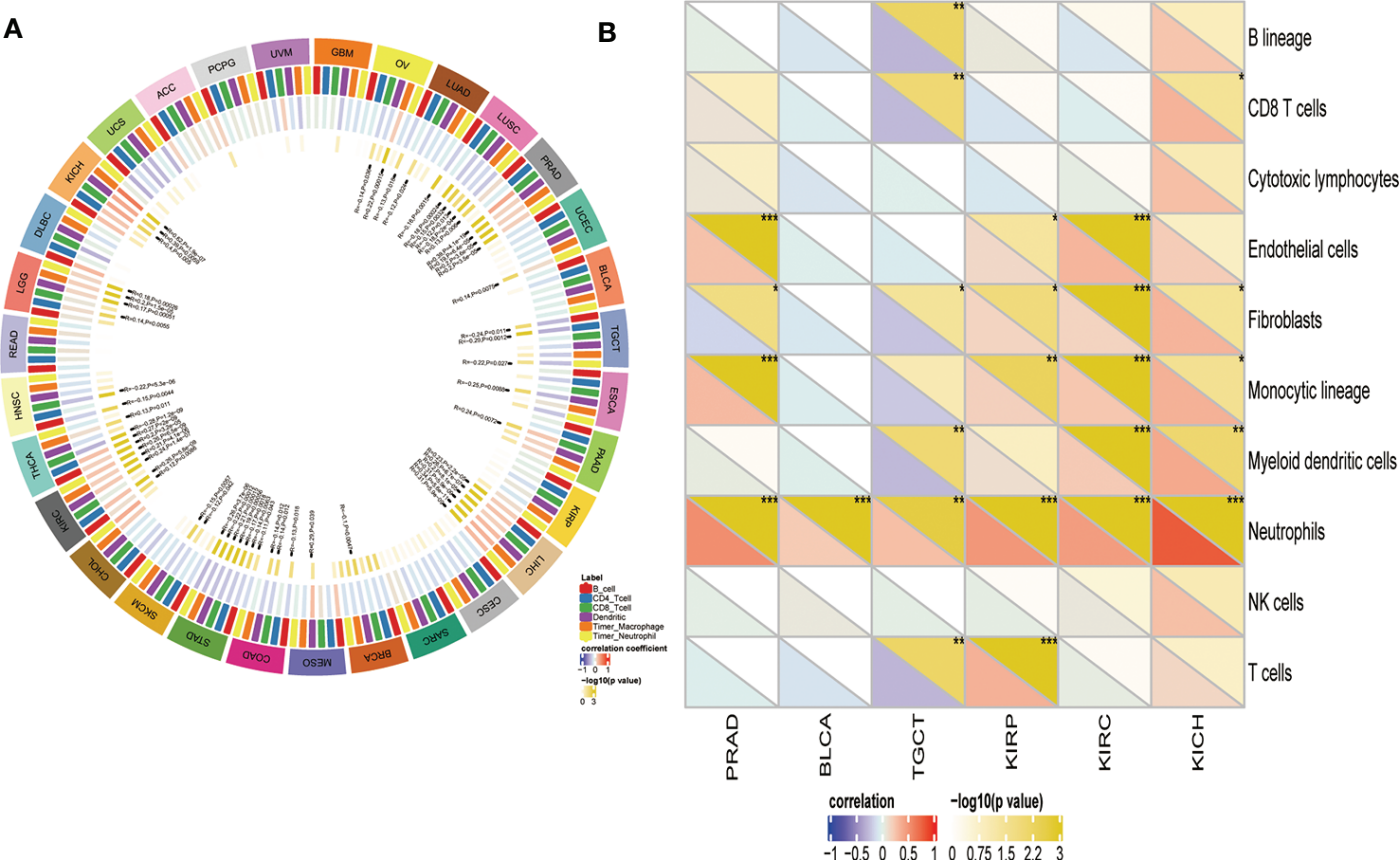
The relationship between YTHDF1 expression and infiltrating immune cells of human cancers and urogenital cancers. **(A)** the relationship between YTHDF1 expression level and infiltrating levels of B cells, CD4+ T cells, CB8+ T cells, macrophages, neutrophils, dendritic cell in human cancers. **(B)** the relationship between YTHDF1 expression level and infiltrating levels of B cell lineages, CB8+ T cells, cytotoxic lymphocytes, endothelial cells, fibroblasts, monocytic cell lineages, myeloid dendritic cells, neutrophils, natural killer cells, T cells in six urogenital cancers. *P < 0.05; **P < 0.01; ***P < 0.001.

After proving the differential YTHDF1 expression in different immune subtypes, we explored the potential correlation between YTHDF1 expression and immune cell infiltration in human cancers, and the results indicated that there was significant correlation in 31 cancer types. YTHDF1 expression had a strong relationship with dendritic cell in 12 cancer types, macrophage in 9 cancer types, neutrophil in 8 cancer types, CD8+ T cells in 13 cancer types, B cells in 10 cancer types and CD4+ T cells in 6 cancer types (**Figure 7A**). The TIMER database’s results consisted of the above results, and all details are shown on **Supplementary Table 4**.

### YTHDF1 Correlates With Immune Cell Infiltration and Their Gene Markers in TME in Urogenital Cancers

Next, we thoroughly analyze the connection between YTHDF1 expression and immune cell infiltration in six urogenital cancers. The results showed that YTHDF1 expression was correlated with the infiltration levels of neutrophils in BLCA, CD8+ T cells, fibroblasts, monocytic cell lineage, myeloid dendritic cells, and neutrophils in KICH; endothelial cells, fibroblasts, monocytic cell lineage, and neutrophils in PRAD; B cell lineage, CB8+ T cells, fibroblasts, myeloid dendritic cells, neutrophils, and T cells in TGCT; endothelial cells, fibroblasts, monocytic cell lineage, neutrophils, and T cells in KIRP and endothelial cells, fibroblasts, monocytic cell lineage, myeloid dendritic cells and neutrophils in KIRC (**Figure 7B**). Next, we verified the above relationship *via* GEPIA and TIMER, and most results from those 2 databases were consistent with the above results. YTHDF1 expression was positively related to immune cells in all urogenital cancers except TGCT. Detailed information on the relationship between YTHDF1 expression and TILs in the GEPIA and TIMER databases is shown in **Supplementary Figures 5**–**10**. In general, the above results strongly identified that YTHDF1 expression influenced immune cell infiltration in urogenital cancers.

Then, we used the TIMER database to further explore the correlation between YTHDF1 expression and different marker subsets of immune cells in six urogenital cancers. The correlation was adjusted by purity to decrease the bias caused by clinical samples. The results showed that YTHDF1 was strongly connected with marker genes of macrophages, Th1 cells, Th2 cells, Tfh cells, Treg cells, and Th17 cells in urogenital cancers. For example, YTHDF1 expression had a strong association with STAT1 in Th1 cells, STAT6 in Th2 cells, STAT3 in Th17 cells, STAT5B in Treg cells, BCL6 in Tfh cells, and IRF5 in M1 macrophages in most cancer types (**Supplementary Table 5**). We used BLCA (n = 408) and PRAD (n = 497) as examples to demonstrate the potential immune function of YTHDF1 in urogenital cancers. According to the results of **Table 1**, YTHDF1 has a close connection with all included marker genes of monocytes, tumor-associated macrophages (TAMs), M2 Macrophages, Th1 cells and regulatory T cells (Tregs) in PRAD. However, YTHDF1 expression in BLCA does not have a strong link since it is only correlated with most marker genes of Th2 cells, and regulatory T cells (Tregs). Different relationships between YTHDF1 expression and gene markers of immune cell infiltration in PRAD and BLCA may explain why there are different survival outcomes in various cancer types. These results again indicate that YTHDF1 expression plays an essential role in the infiltration of immune cells in human cancers, and that YTHDF1 might regulate the function of immune cells by participating in a mechanism similar to that of marker genes.

**Table 1:** Correlation between YTHDF1 and relate genes and markers of immune cells analyzed by TIMER.

Description	Gene markers	PRAD				BLCA			
		None		Purity		None		Purity	
		Cor	P	Cor	P	Cor	P	Cor	P
CD8+ T cell	CD8A	0.197	***	0.220	***	−0.044	0.380	0.015	0.772
	CD8B	0.141	**	0.134	**	−0.002	0.966	0.026	0.614
T cell (general)	CD3D	−0.011	0.808	−0.041	0.407	−0.124	*	−0.087	0.096
	CD3E	0.087	0.052	0.079	0.106	−0.077	0.120	−0.015	0.779
	CD2	0.119	**	0.089	0.070	−0.054	0.278	0.012	0.824
B cell	CD19	−0.041	0.356	−0.058	0.235	0.014	0.771	0.093	0.075
	CD79A	0.041	0.366	0.014	0.782	−0.051	0.304	0.011	0.833
Monocyte	CD86	0.260	***	0.226	***	−0.009	0.859	0.088	0.090
	CD115 (CSF1R)	0.229	***	0.193	***	−0.048	0.330	0.034	0.516
TAM	CCL2	0.156	***	0.168	***	0.006	0.900	0.109	*
	CD68	0.219	***	0.195	***	−0.030	0.546	0.007	0.901
	IL10	0.274	***	0.241	***	−0.018	0.724	0.088	0.091
M1 Macrophage	INOS (NOS2)	−0.097	*	−0.046	0.348	0.115	*	0.100	0.056
	IRF5	0.298	***	0.337	***	0.120	*	0.125	*
	COX2(PTGS2)	0.304	***	0.290	***	0.047	0.339	0.077	0.139
M2 Macrophage	CD163	0.310	***	0.276	***	−0.024	0.622	0.073	0.160
	VSIG4	0.251	***	0.220	***	−0.066	0.187	0.013	0.800
	MS4A4A	0.217	***	0.196	***	−0.052	0.292	0.037	0.476
Neutrophils	CD66b (CEACAM8)	0.031	0.490	0.047	0.339	0.122	*	0.092	0.079
	CD11b (ITGAM)	0.221	***	0.204	***	−0.015	0.761	0.085	0.105
	CCR7	0.158	***	0.149	**	0.039	0.431	0.096	0.066
Natural killer cell	KIR2DL1	0.101	*	0.109	*	−0.050	0.317	−0.020	0.698
	KIR2DL3	0.073	0.106	0.086	0.079	−0.065	0.187	−0.029	0.585
	KIR2DL4	0.234	***	0.258	***	−0.058	0.243	−0.019	0.717
	KIR3DL1	0.102	*	0.108	*	−0.040	0.418	−0.010	0.847
	KIR3DL2	0.092	*	0.062	0.209	−0.025	0.616	0.006	0.915
	KIR3DL3	0.000	0.997	−0.016	0.738	−0.023	0.645	−0.022	0.678
	KIR2DS4	0.030	0.499	0.039	0.430	−0.034	0.494	−0.003	0.952
Dendritic cell	HLA-DPB1	−0.005	0.904	−0.005	0.914	−0.051	0.304	0.028	0.596
	HLA-DQB1	0.095	*	0.086	0.079	−0.025	0.609	0.054	0.299
	HLA-DRA	0.214	***	0.216	***	0.009	0.863	0.095	0.069
	HLA-DPA1	0.200	***	0.181	***	−0.010	0.835	0.069	0.184
	BDCA-1(CD1C)	0.185	***	0.169	***	−0.069	0.163	−0.023	0.662
	BDCA-4(NRP1)	0.529	***	0.522	***	0.061	0.220	0.090	0.085
	CD11c (ITGAX)	0.218	***	0.233	***	−0.015	0.763	0.093	0.075
Th1	T-bet (TBX21)	0.135	**	0.161	***	−0.034	0.489	0.018	0.731
	STAT4	0.172	***	0.168	***	−0.032	0.525	0.029	0.580
	STAT1	0.557	***	0.546	***	0.157	**	0.223	***
	IFN-γ (IFNG)	0.139	**	0.120	*	−0.026	0.596	0.018	0.736
	TNF-α (TNF)	0.194	***	0.177	***	0.103	*	0.154	**
	IL12A	0.104	*	0.111	*	−0.047	0.341	0.016	0.762
	IL12B	0.173	**	0.118	*	−0.067	0.174	−0.018	0.733
Th2	GATA3	0.096	*	0.099	*	0.155	**	0.137	**
	STAT6	0.264	***	0.239	***	0.183	***	0.176	***
	STAT5A	0.166	***	0.166	***	0.048	0.338	0.099	0.057
	IL13	0.062	0.170	0.092	0.061	−0.126	*	−0.109	*
Tfh	BCL6	0.304	***	0.351	***	0.163	***	0.152	**
	IL21	0.084	0.061	0.088	0.073	0.039	0.429	0.089	0.089
Th17	STAT3	0.639	***	0.629	***	0.255	***	0.302	***
	IL17A	0.065	0.146	−0.008	0.866	0.033	0.512	0.047	0.372
Treg	FOXP3	0.274	***	0.300	***	0.074	0.135	0.166	**
	CCR8	0.370	***	0.340	***	0.144	**	0.234	***
	STAT5B	0.468	***	0.463	***	0.281	***	0.279	***
	TGFβ (TGFB1)	0.134	**	0.179	***	−0.122	*	−0.145	**
T cell exhaustion	PD-1 (PDCD1)	0.033	0.459	0.061	0.212	−0.053	0.283	0.007	0.897
	CTLA4	0.044	0.323	0.047	0.338	−0.050	0.317	0.013	0.801
	LAG3	−0.124	**	−0.111	*	−0.040	0.419	0.021	0.692
	TIM-3 (HAVCR2)	0.235	***	0.227	***	−0.029	0.555	0.069	0.186
	GZMB	0.122	**	0.143	**	−0.115	*	−0.057	0.273

PRAD, prostate adenocarcinoma; BLCA: bladder urothelial carcinoma; TAM, tumor-associated macrophage; Th, T helper cell; Tfh, Follicular helper T cell; Treg, regulatory T cell; Cor, R value of Spearman’s correlation; None, correlation without adjustment. Purity, correlation adjusted by purity. *P < 0.05; **P < 0.01; ***P < 0.001.

### YTHDF1 Gene and Expression Altered in Different Clinical Subgroups of Urogenital Cancers

**Figure 8:**
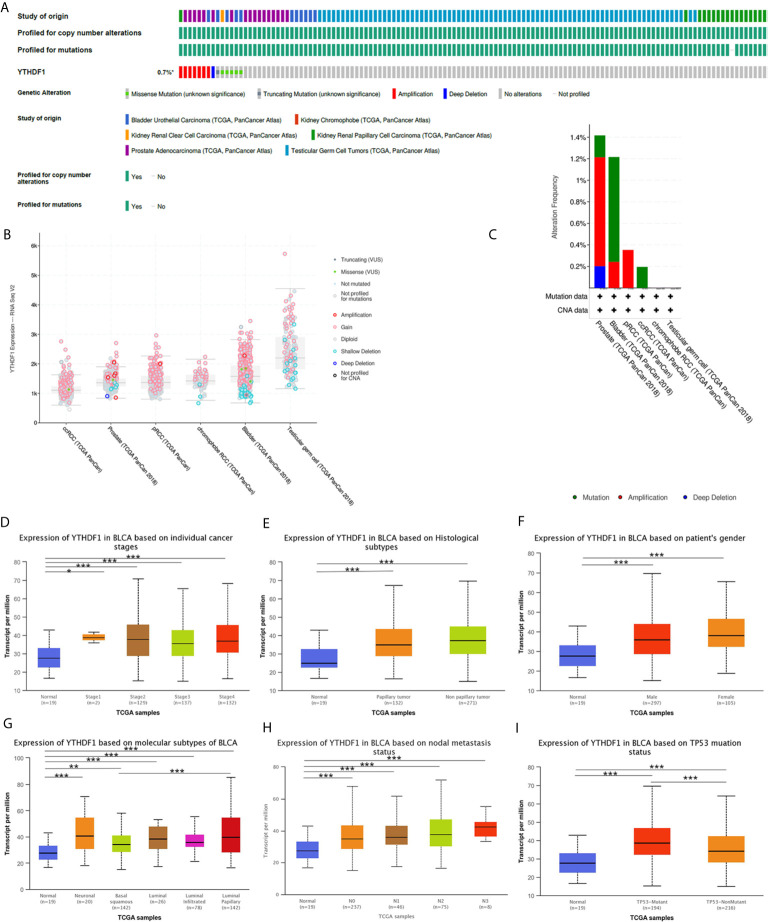
YTHDF1 genomic alterations in six urogenital cancers analyzed by the cBioPortal database **(A–C)** and YTHDF1 differential expression in bladder cancer with different clinical subgroups **(D–I)** analyzed by the UALCAN database. **(A)** OncoPrint of YTHDF1 gene alterations in cancer cohort. (Different colors means different types of genetic alterations and amplification accounts for the largest proportion). **(B)** main type of YTHDF1 gene alterations in cancer groups. **(C)** Details of YTHDF1 gene alteration types in cancer cohort; **(D–I)** YTHDF1 differential expression in bladder cancer with individual cancer stages (n = 419) **(D)**, histological subtypes (n = 422) **(E)**, patient sex (n = 421) **(F)**, molecular subtypes (n = 427) **(G)**, nodal metastasis status (n = 385) **(H)**, TP53 mutation status (n = 429) **(I)**. (*P < 0.05, **P < 0.01, ***P < 0.001).

Analysis of YTHDF1 genomic alterations in urogenital cancers was explored with the cBioPortal website. The results showed that genomic alterations of YTHDF1 occurred in 0.7% of patients (**Figure 8A**). The types of YTHDF1 gene alterations were diverse, resulting in changes in gene expression (**Figure 8B**). Copy number variation (CNV) mainly occurred in PRAD and BLCA, while no CNV occurred in KICH and TGCT (**Figure 8C**). Next, we used the UALCAN database to explore YTHDF1 expression in urogenital cancers with different clinical characteristics. Taking BLCA as an example, we found that YTHDF1 was significantly differentially expressed existed in different cancer stages, histological subtypes, patient sex, molecular subtypes, nodal metastasis status and TP53 mutation status of BLCA (**Figures 8D–I**). Additionally, differential YTHDF1 expression in another five urogenital cancers with different clinical characteristics is displayed in **Supplementary Figure 11**. All the above results suggested that YTHDF1 genomic alteration and differential expression indeed occur in cancer tissue, and might play an essential role in cancer onset and progression.

### YTHDF1 Coexpression Networks Correlate With the Immune Response

**Figure 9:**
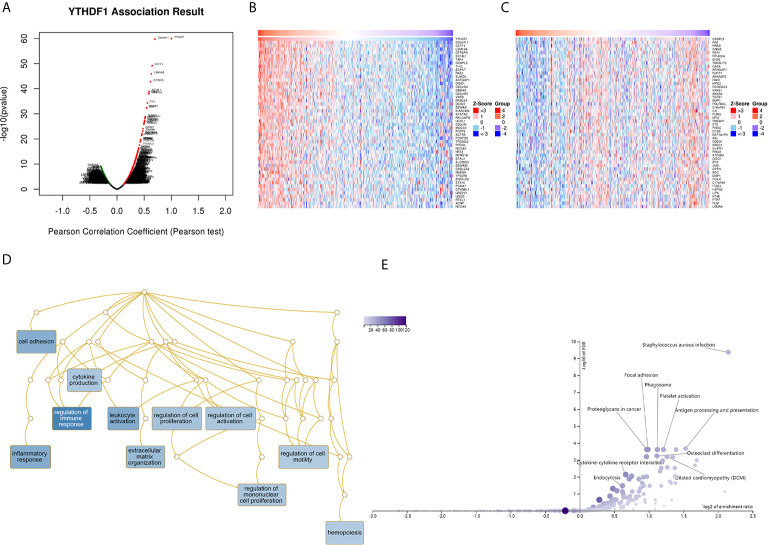
YTHDF1 coexpression genes in BLCA analyzed by the LinkedOmics database. **(A)** Highly correlated genes of YTHDF1 tested by Pearson test in BLCA cohort. **(B, C)** Top 50 positive coexpression genes **(B)** and negative coexpression genes **(C)** of YTHDF1 in heat map in BLCA; **(D)** Directed acyclic graph of YTHDF1 GO analysis (biological process) in BLCA cohort. **(E)** Volcano plot of YTHDF1 KEGG pathways in BLCA cohort.

The above results identified that YTHDF1 had a significant association with the prognosis and immunity of cancers. Next, we explored YTHDF1 coexpression networks using the LinkedOmics database to verify the potential function of YTHDF1 in tumor tissue, and used BLCA as an example to illustrate the potential effect. In BLCA, 1396 genes (dark red dots) were significantly positively related to YTHDF1, and 1143 genes (dark green dots) were negatively related (false discovery rate (FDR) < 0.01) (**Figure 9A**). A heat map was used to display the top 50 genes positively and negatively correlated with YTHDF1 (**Figures 9B, C**). Moreover, all coexpressed genes were displayed in **Supplementary Table 6**. C20orf11, CSTF1, and LSM14B (r = 0.631) had the strongest association with YTHDF1 expression (r = 0.696, 0.647, 0.631 and P = 1.90E-60, 9.72E-46, 6.43E-46, respectively). Next, gene set enrichment analysis (GSEA) was used to determine the main gene ontology (GO) terms of YTHDF1 coexpression genes. We further analyzed the biological process categories of GO and found that YTHDF1 and its co-expression genes primarily participated in the regulation of immune response, leukocyte activation and inflammatory response (**Figure 9D**). Then, we carried out Kyoto Encyclopedia of Genes and Genomes (KEGG) pathway analysis, and the results showed that coexpressed genes were enriched in staphylococcus aureus infection, antigen processing and presentation, and cytokine-cytokine receptor interaction (**Figure 9E**). These results suggested that YTHDF1 expression might play an essential role in human cancers by regulating the immune response of the TME.

## Discussion

YTHDF1, a “reader” of m6A, impairs the activation of DCs and CD8+ T cells by increasing the translation of lysosomal cathepsins and thus degrading tumor neoantigens (4, 17). Moreover, in mice with low YTHDF1 expression, there was a higher level of CD8+ T cells and NK cells in the TME, which means enhanced immunosurveillance in the absence of YTHDF1 (17). Moreover, YTHDF1 also plays an important role in oncogenesis and tumor progression in various cancers. For example, YTHDF1 promotes the growth and progression of bladder cancer *via* the ITGA6-METTL3 pathway (43). In colorectal cancer, YTHDF1 plays a vital oncogenic role in cell self-renewal and differentiation *via* the Wnt/β-catenin pathway (44). In addition, YTHDF1 also produces a marked effect on lung cancer growth and metastasis by mediating YAP expression and activity (14). Although anti-programmed death 1 (PD-1)/programmed death ligand-1(PD-L1) drugs have achieved considerable clinical efficacy and durable response with low toxicity (45, 46), they are not effective for all cancer types or do not achieve ideal efficacy in all patients (47). A new study identified that YTHDF1 deficiency in classical dendritic cells enhanced the cross-presentation of tumor antigens and the cross-priming of CD8+ T cells, and the therapeutic efficacy of the anti-PD-L1 drug was improved after blocking YTHDF1 (17). The YTHDF1 inhibitor GB1107 was also proven to inhibit the growth and metastasis of lung adenocarcinoma *in vivo* (48). Consistent with our findings and hypothesis, GB1107 increased tumor M1 macrophage polarization and CD8+ T cell infiltration and potentiated the effects of a PD-L1 immune checkpoint inhibitor (48). Therefore, YTHDF1 can be used as a new anticancer immunotherapy drug target or in combination with known immune checkpoint inhibitors to enhance immune infiltration and responses in cancers. These studies indicated that YTHDF1 might be a promising target in antitumor immunotherapy. Furthermore, combining anti-PD-L1 drugs with YTHDF1 depletion could be used as a new antitumor strategy.

In the first step of our study, we used the Oncomine, TIMER, GEPIA, and BioGPS databases to determine the expression level of YTHDF1 in cancers and normal tissues. The results showed that YTHDF1 had significantly higher expression in most cancer types except lymphoma and THCA, which was consistent with previous studies in lung and colorectal cancer (14, 44). These results indicated that YTHDF1 indeed promotes oncogenesis and tumor progression in human cancers.

Next, the relationship between YTHDF1 expression and prognosis was explored. Similar to previous studies, high YTHDF1 expression patients had a worse prognosis in LIHC and TCGT (49, 50). In addition, in many other cancer types such as ACC, BLCA, MESO, UVM, LIHC, and UCS, high YTHDF1 expression meant worse prognosis, which proved that YTHDF1 was a potential prognostic pan-cancer biomarker. Then, we explored YTHDF1 expression in different immune subtypes and molecular subtypes of human cancers to determine its potential mechanism of action. The results showed that YTHDF1 expression was significantly different in different immune subtypes and molecular subtypes in most cancer types, which might prove that YTHDF1 is a promising diagnostic pan-cancer biomarker and participates in immune regulation. Moreover, we demonstrated that there were significant differences in YTHDF1 expression in different clinical subgroups of urogenital cancers, consistent with a previous study showing that YTHDF1 is expressed differently in KIRC at different stages (50). In fact, YTHDF1 differential YTHDF1 expression existed in all urogenital cancers with diverse clinical characteristics, which indicated that YTHDF1 might play a role in the growth and progression of cancers.

Tumor-infiltrating lymphocytes (TILs) in the TME have been proven to be an independent predictor of cancer patient prognosis and immunotherapeutic efficacy (51, 52). Our study demonstrated that YTHDF1 had a strong association with TILs and played an essential role in the TME. For example, YTHDF1 expression was strongly significantly related to CD8+T cells, macrophages and dendritic cells. As a universal component of the TME, macrophages have been proven to contribute to immune evasion and suppression (53). The TME with low YTHDF1 expression contained higher levels of CD8+ T cells and natural killer (NK) cells in a mouse model, and enhanced the ability to DCs in cross-present of tumor antigens (17). In particular, in PRAD, we found that TYHDF1 expression has a close association with TILs in the TME. For example, YTHDF1 expression has a close association with IFN-gamma in PRAD, which is regarded as a hallmark of antitumor immunity (54). Moreover, YTHDF1 expression is related to all of the examined marker genes of CD8+ T cells, monocytes, tumor-associated macrophages (TAMs), M2 macrophages, Th1 cells, and other known immune stimulatory and immune suppressive cytokines, which proves the potential immune function of YTHDF1 in PRAD.

Our study also found that YTHDF1 was negatively related to the immune, stromal and ESTIMATE scores of the TME in most human cancer types, but YTHDF1 expression was not always negatively related to TILs in human cancers, which indicated that YTHDF1 plays a different immune regulation role in various cancer types. The correlation between YTHDF1 and tumor mutational burden (TMB), microsatellite instability (MSI), and neoantigens also proved that YTHDF1 closely related with the TME in human cancers. However, further experimental research to prove its function.

In our analysis of YTHDF1 coexpression networks, we found that YTHDF1 and its coexpression genes indeed took part in regulating the immune response and antigen processing and presentation. Furthermore, a previous study proved that anti-PD-L1 drugs achieved better therapeutic efficacy in YTHDF1 low expression conditions (17). A strong relationship between YTHDF1 and ICP genes was found, which provides a theoretical basis for combined molecular targeting immunotherapy for the future. In summary, the above results strongly indicated the potential of YTHDF1 as a target in anticancer immunotherapy.

However, even though we performed a comprehensive and systematic analysis on YTHDF1 and used different databases and R 3.2.2 for cross-verification, there are some limitations to this study. First, the microarray and sequencing data from different databases exhibited differences and lacked granularity and specificity, which might cause systematic bias. Second, *in vivo*/*in vitro* experiments are needed to prove our results on the potential function of YTHDF1, which could increase the credibility of our results. Third, even though we concluded that YTHDF1 expression was strongly related to immune cell infiltration and prognosis of human cancers, we lack direct evidence on YTHDF1 influencing prognosis by taking part in immune infiltration, Therefore, the mechanisms by which YTHDF1 participate in immune regulation remain unknown, and the exact pathway needs further study. Fourth, no anti-YTHDF1 therapeutic monoclonal antibodies have been evaluated in clinical trials. Therefore, we have no specific and complete cases with data to identify the benefit of anti-YTHDF1 targeting drugs in the survival of cancer models or inhibiting tumor growth. In the future, a prospective study of YTHDF1 expression and its role in human cancers’ immune infiltration is needed, along with successful development and testing of a new antitumor immunotherapy drug targeting YTHDF1.

## Data Availability Statement

The datasets presented in this study can be found in online repositories. The names of the repository/repositories and accession number(s) can be found in the article/**Supplementary Material**.

## Author Contributions

JianH, DQ, AY and JiaoH were responsible for experimental design, experimental analysis and thesis writing. HD, HL, and ZY were responsible for data screening, collection and writing. JC and XZ were responsible for the guidance and review of the thesis. All authors contributed to the article and approved the submitted version.

## Funding

We thank the National Natural Science Foundation of China (81902592), Hunan Province Key R&D Program (2019SK2202) and Xiangya Hospital Youth Fund (2018Q09) for the financial support. This article has not received sponsorship for publication.

## Conflict of Interest

The authors declare that the research was conducted in the absence of any commercial or financial relationships that could be construed as a potential conflict of interest.

## Supplementary Material

Supplementary Figure 1 YTHDF1 expression in different cancer cell lines **(A)** and normal tissue **(B)** investigated by the BioGPS database.

Supplementary Figure 2 Kaplan-Meier survival curve of human cancers with high and low YTHDF1 expression analyzed by the Kaplan-Meier plotter database. **(A–G)** Overall survival of ESCA, HNSC, PPAD, PCPG, READ, THCA, and UCEC with high and low YTHDF1 expression (n = 80, 499, 177, 178, 165, and 502 respectively); **(H–N)** Progression-free survival of ESSC, HNSC, LUAD, PAAD, READ, STAD, and UCEC with high and low YTHDF1 expression (n = 54, 124, 300, 69, 47, 215, and 422, respectively).

Supplementary Figure 3 Relationship between YTHDF1 expression and pan-cancer immune subtypes.

Supplementary Figure 4 Relationship between YTHDF1 expression and pan-cancer molecular subtypes.

Supplementary Figure 5 Correlation between YTHDF1 expression and 24 tumor infiltrating lymphocytes (TILs) in KICH analyzed by the GEPIA database.

Supplementary Figure 6 Correlation between YTHDF1 expression and 24 tumor infiltrating lymphocytes (TILs) in PRAD analyzed by the GEPIA database.

Supplementary Figure 7 Correlation between YTHDF1 expression and 24 tumor infiltrating lymphocytes (TILs) in KIRC analyzed by the GEPIA database.

Supplementary Figure 8 Correlation between YTHDF1 expression and 24 tumor infiltrating lymphocytes (TILs) in KIRP analyzed by the GEPIA database.

Supplementary Figure 9 Correlation between YTHDF1 expression and 24 tumor infiltrating lymphocytes (TILs) in BLCA analyzed by the GEPIA database.

Supplementary Figure 10 Correlation between YTHDF1 expression and 24 tumor infiltrating lymphocytes (TILs) in TGCT analyzed by the GEPIA database.

Supplementary Figure 11 YTHDF1 differential expression in five urogenital cancers with different clinical subgroups analyzed by the UALCAN database.

Supplementary Table 1 Sample Numbers of the TCGA Cancer types and corresponding immune and molecular subtypes.

Supplementary Table 2 Correlation between YTHDF1 expression and survival in the GEO datasets analyzed by the PrognoScan database.

Supplementary Table 3 Detailed relationship between YTHDF1 expression and pan-cancer immune cell infiltration in the tumor microenvironment.

Supplementary Table 4 Relationship between YTHDF1 expression and related immune marker sets of urogenital cancers analyzed by the TIMER database.

Supplementary Table 5 24 tumors associated infiltrating lymphocytes (TILs) and their gene markers.

Supplementary Table 6 YTHDF1 coexpression genes in urogenital cancers analyzed by the LinkedOmics database.

## Abbreviations

YTHDF1, YTH N6-methyladenosine RNA binding protein 1; ICP, immune checkpoints; TMB, tumor mutational burden; MSI: microsatellite instability; TILs, tumor immune-infiltrated cells; m6A, N6-methyladenine; TCGA, the Cancer Genome Atlas; ESTIMATE, Estimation of Stromal and Immune cells in Malignant Tumor tissues using Expression data; OS, overall survival; DFS, disease-free survival; RFS, relapse-free survival; ACC, adrenocortical carcinoma; BLCA, bladder urothelial carcinoma; BRCA, breast invasive carcinoma; CESC, cervical squamous cell carcinoma and endocervical adenocarcinoma; COAD, colon adenocarcinoma; READ, colon adenocarcinoma; ESCA, esophageal carcinoma; GBM, glioblastoma multiforme; HNSC, head and neck squamous cell carcinoma; KICH, kidney chromophobe; KIRC, kidney renal clear cell carcinoma; KIRP, kidney renal papillary cell carcinoma; LAML, acute myeloid leukemia; LGG, brain lower grade glioma; LIHC, liver hepatocellular carcinoma; LUAD, lung adenocarcinoma; LUSC, lung squamous cell carcinoma; MESO, mesothelioma; OV, ovarian serous cystadenocarcinoma; PAAD, pancreatic adenocarcinoma; PCPG, pheochromocytoma and paraganglioma; PRAD, prostate adenocarcinoma; READ, rectum adenocarcinoma; STAD, stomach adenocarcinoma; TGCT, testicular germ cell tumors; THCA, thyroid carcinoma; UCEC, uterine corpus endometrial carcinoma; UCS, uterine carcinosarcoma; TME, tumor microenvironment.
