# Supplementary figures and images for: YTHDF1 Is a Potential Pan-Cancer Biomarker for Prognosis and Immunotherapy
Source: Front Oncol. 2021 May 6;11:607224. doi: 10.3389/fonc.2021.607224 (PMC8134747; doi:10.3389/fonc.2021.607224)

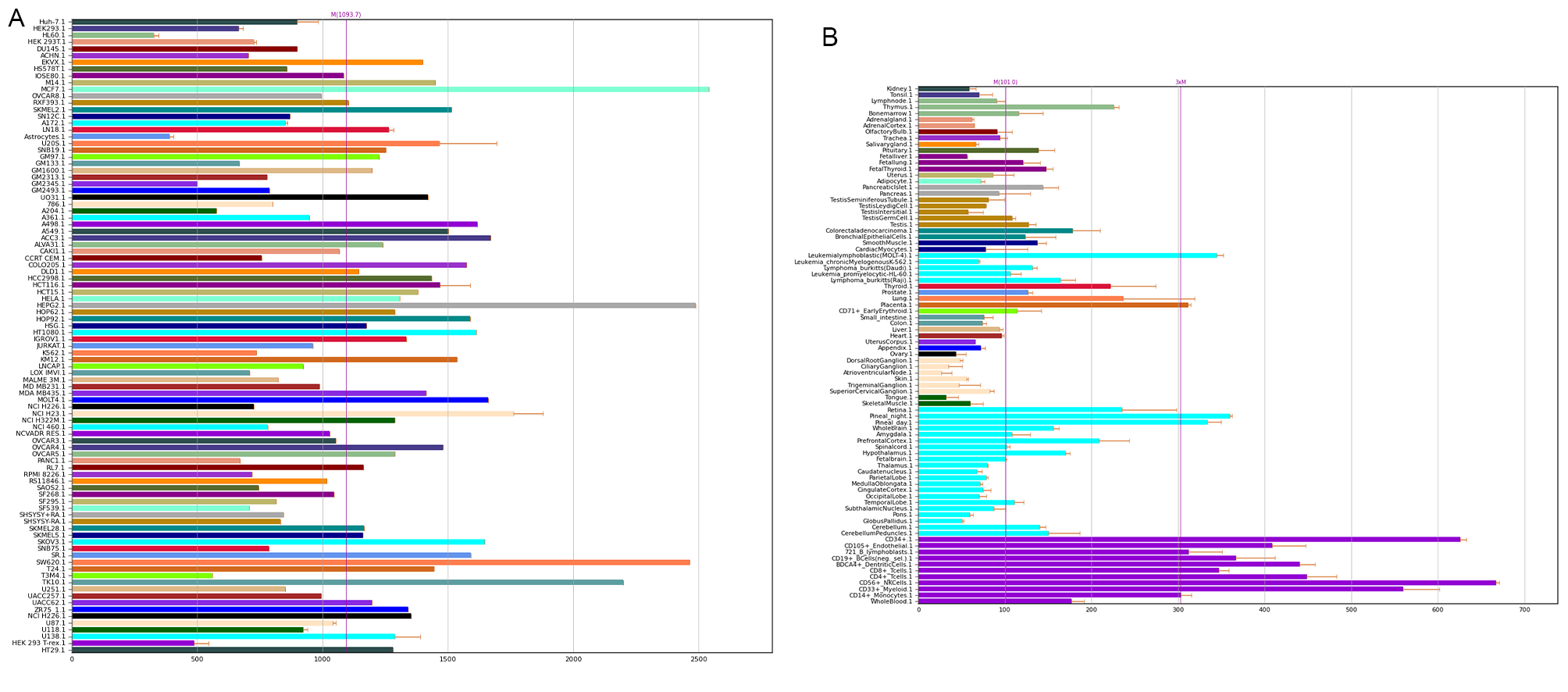

Supplement: Supplementary Figure 1 — YTHDF1 expression in different cancer cell lines (A) and normal tissue (B) investigated by the BioGPS database. [file Image_1.tif]

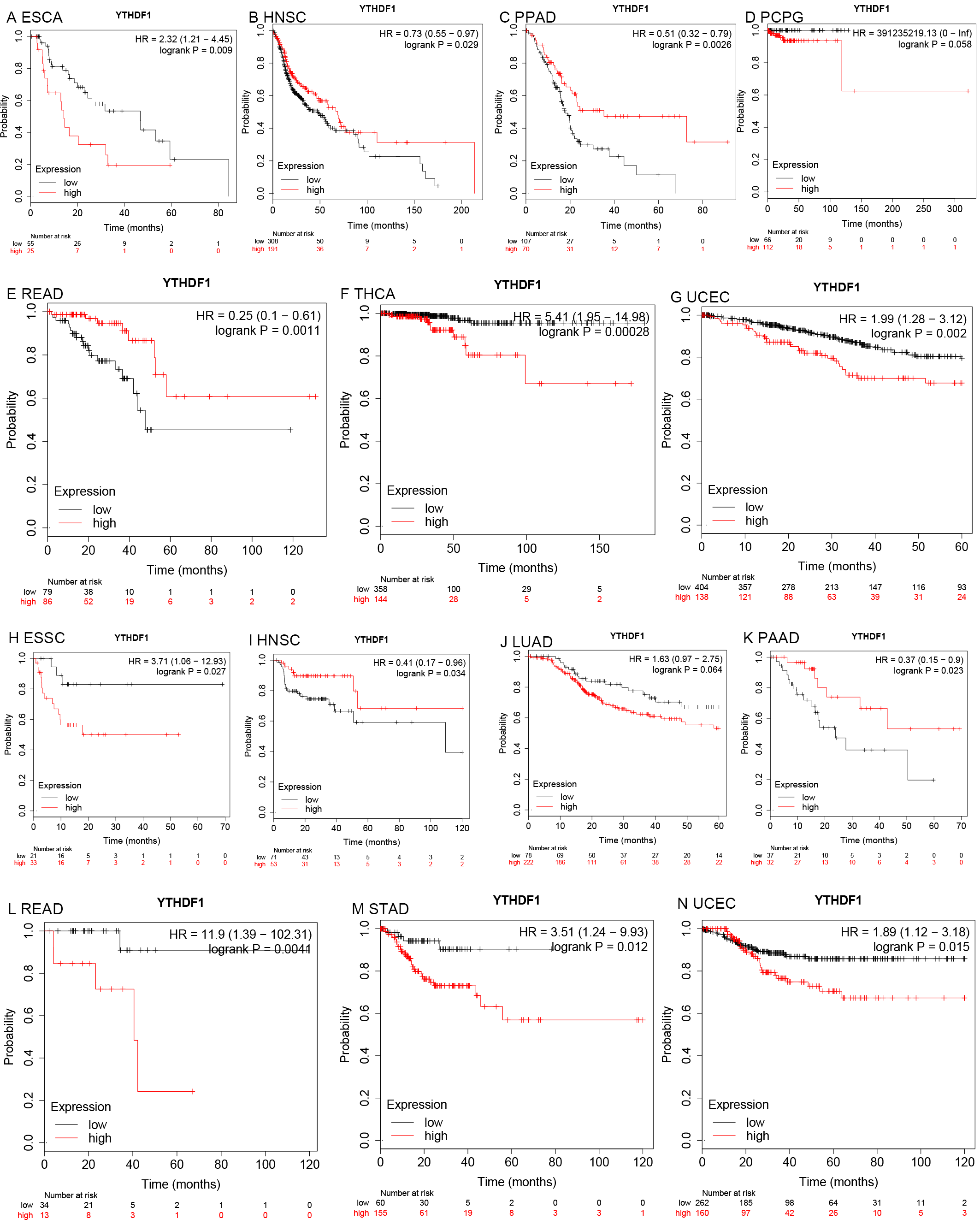

Supplement: Supplementary Figure 2 — Kaplan-Meier survival curve of human cancers with high and low YTHDF1 expression analyzed by the Kaplan-Meier plotter database. (A–G) Overall survival of ESCA, HNSC, PPAD, PCPG, READ, THCA, and UCEC with high and low YTHDF1 expression (n = 80, 499, 177, 178, 165, and 502 respectively); (H–N) Progression-free survival of ESSC, HNSC, LUAD, PAAD, READ, STAD, and UCEC with high and low YTHDF1 expression (n = 54, 124, 300, 69, 47, 215, and 422, respectively). [file Image_2.tif]

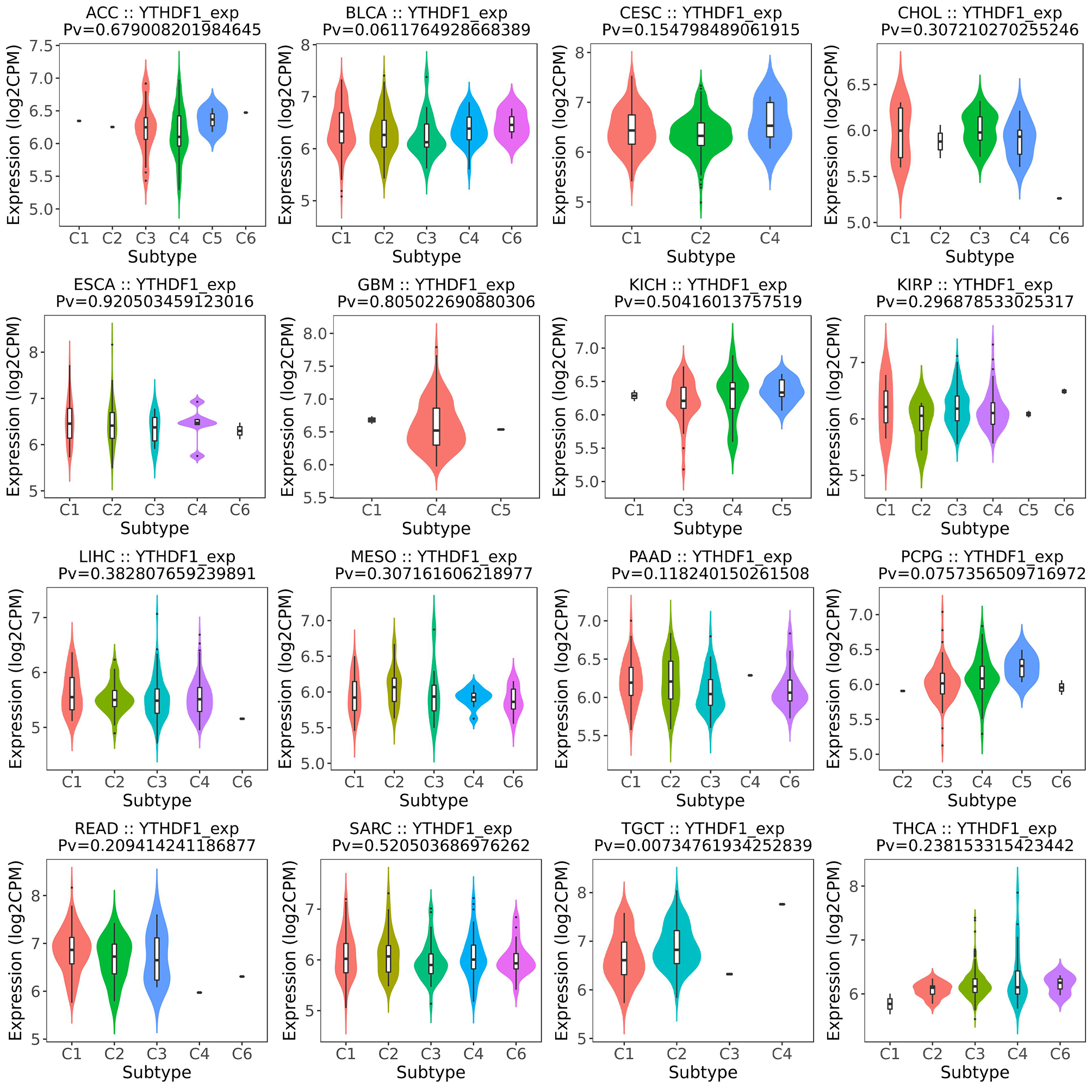

Supplement: Supplementary Figure 3 — Relationship between YTHDF1 expression and pan-cancer immune subtypes. [file Image_3.tif]

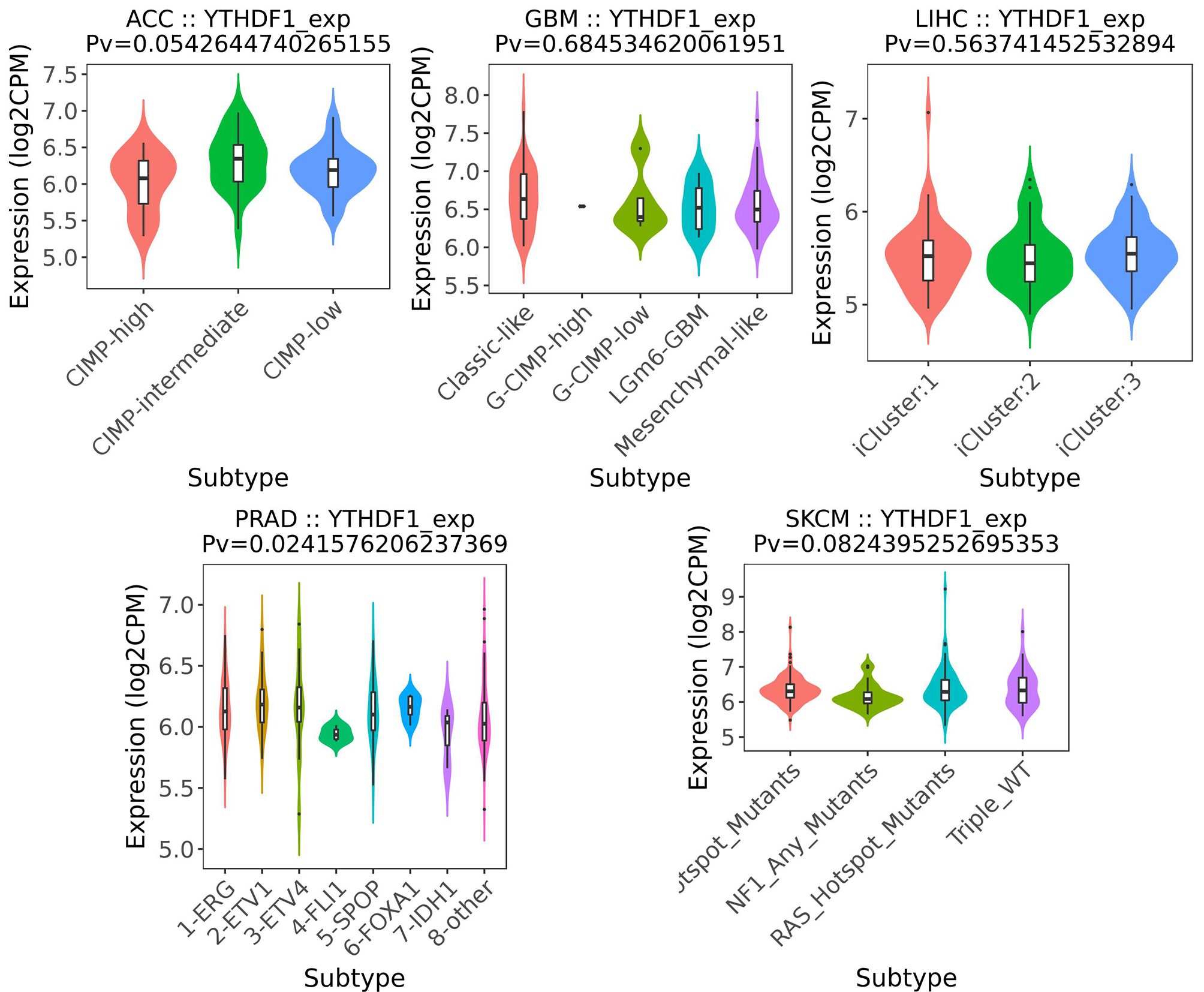

Supplement: Supplementary Figure 4 — Relationship between YTHDF1 expression and pan-cancer molecular subtypes. [file Image_4.tif]

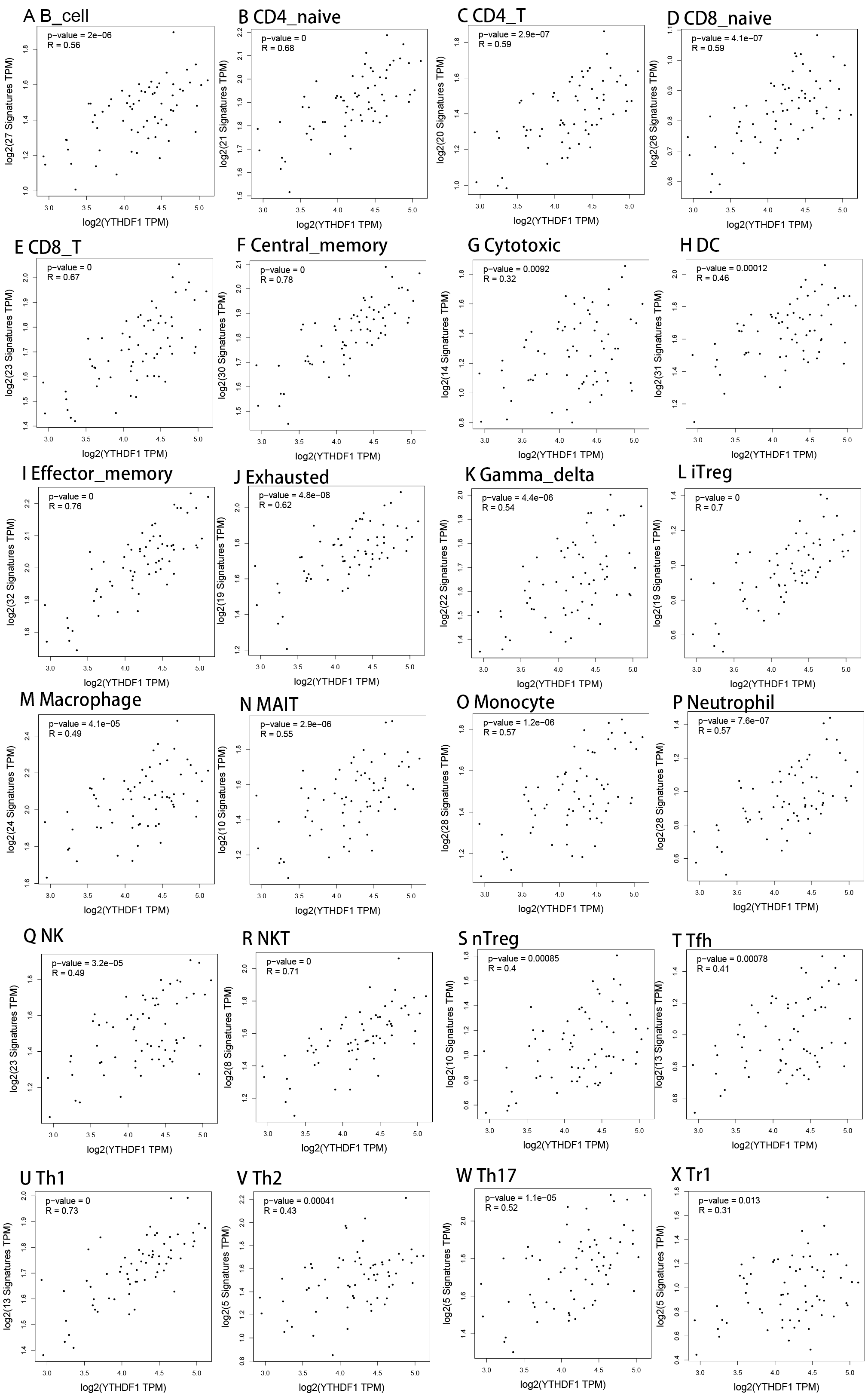

Supplement: Supplementary Figure 5 — Correlation between YTHDF1 expression and 24 tumor infiltrating lymphocytes (TILs) in KICH analyzed by the GEPIA database. [file Image_5.tif]

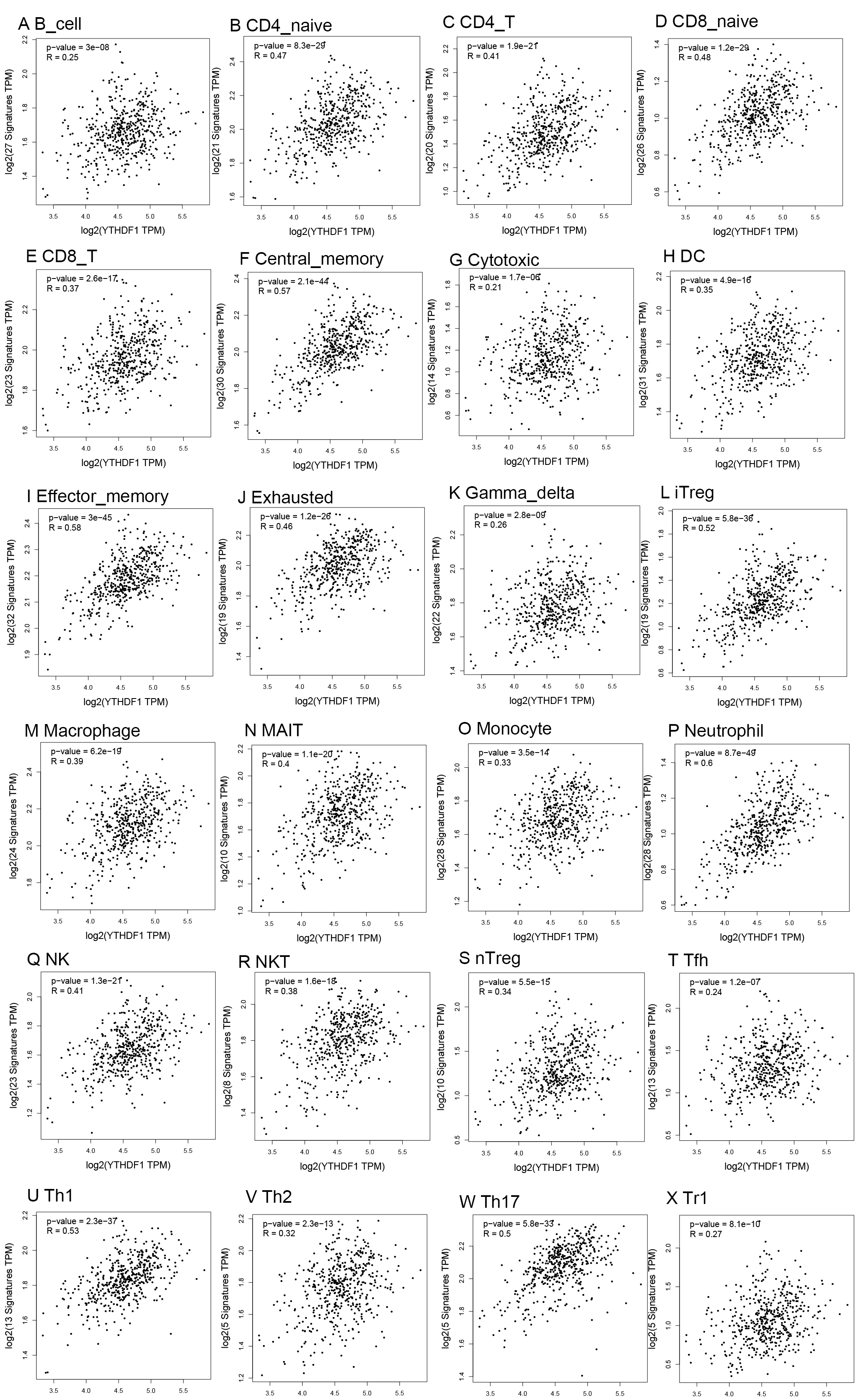

Supplement: Supplementary Figure 6 — Correlation between YTHDF1 expression and 24 tumor infiltrating lymphocytes (TILs) in PRAD analyzed by the GEPIA database. [file Image_6.tif]

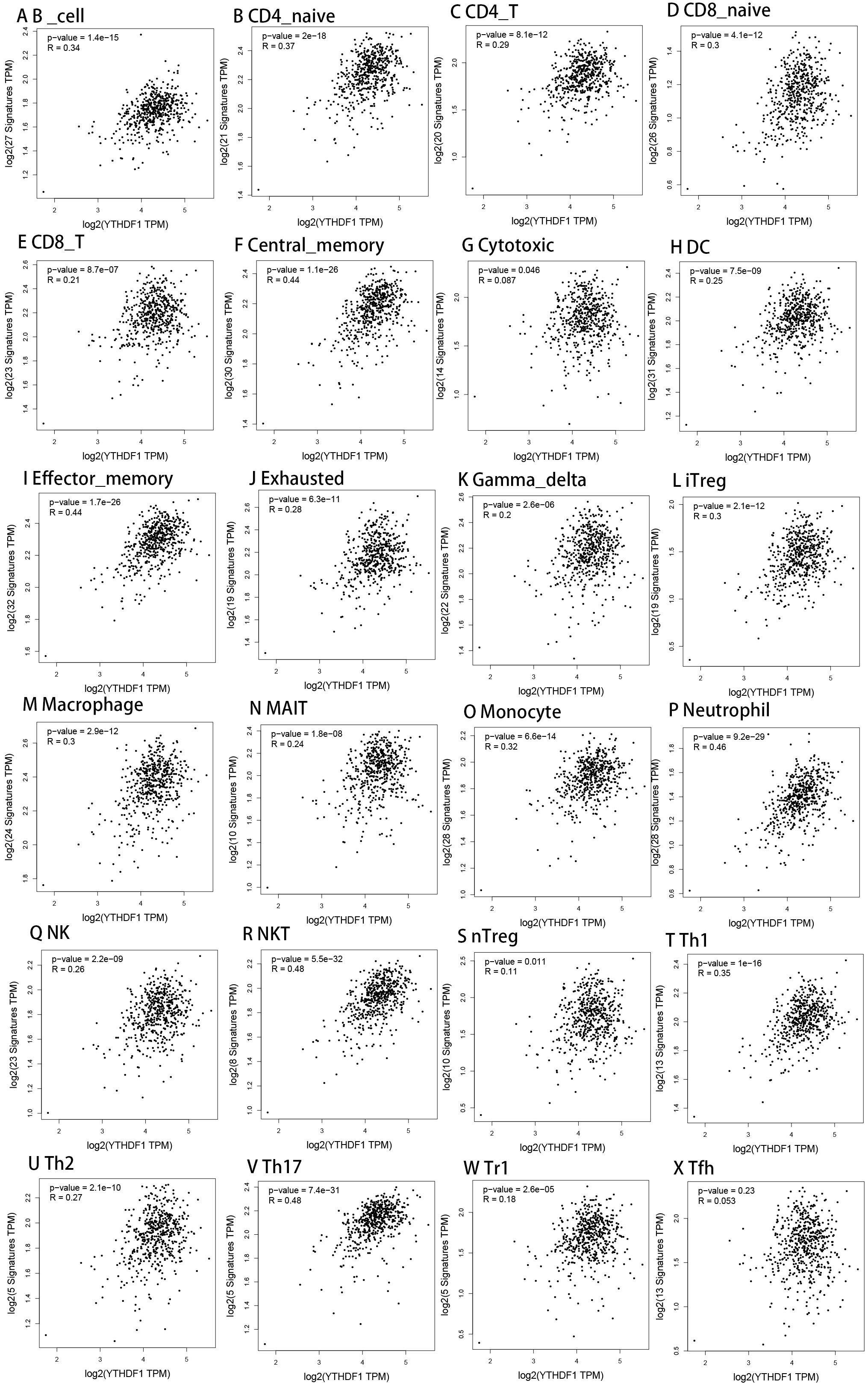

Supplement: Supplementary Figure 7 — Correlation between YTHDF1 expression and 24 tumor infiltrating lymphocytes (TILs) in KIRC analyzed by the GEPIA database. [file Image_7.tif]

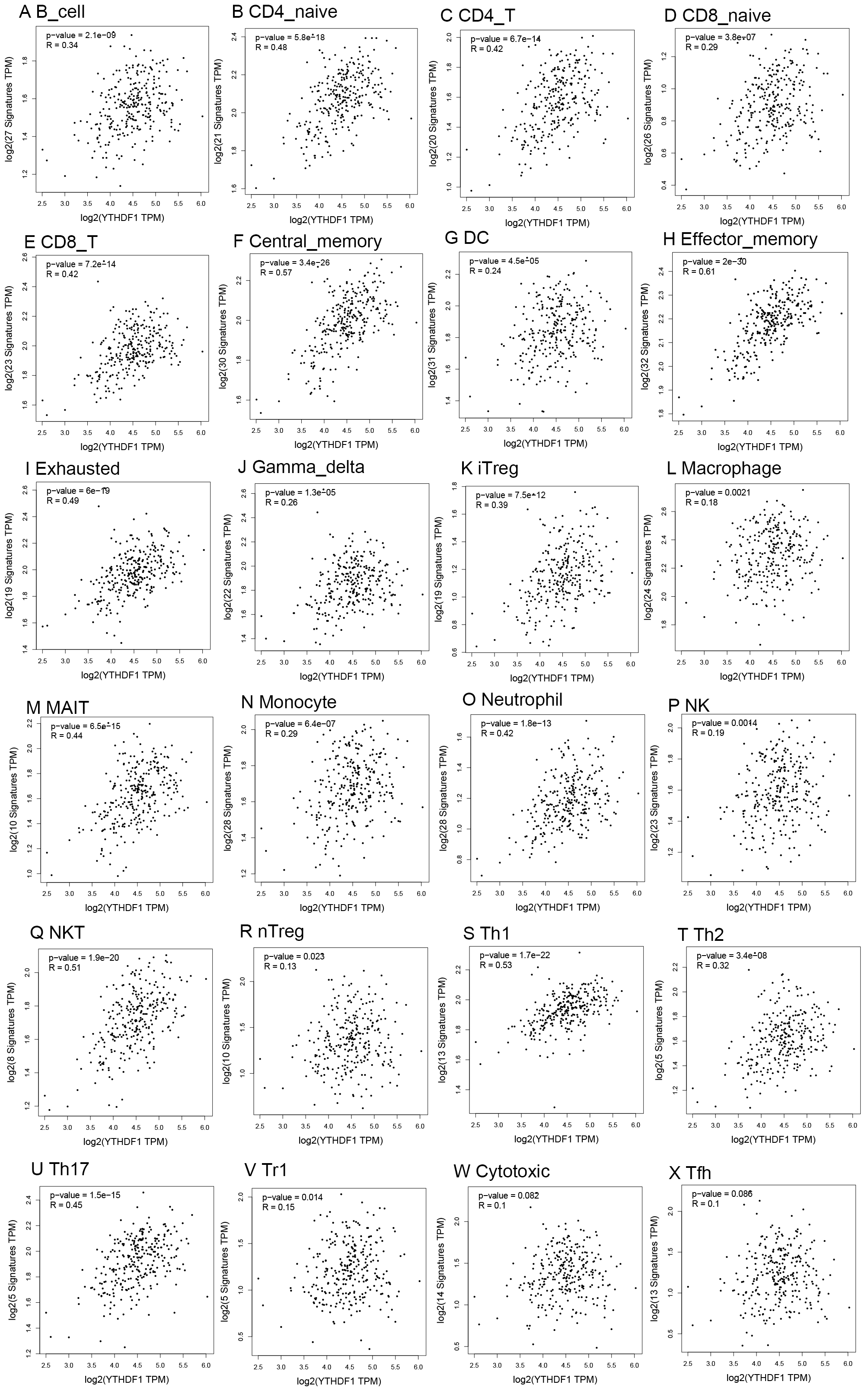

Supplement: Supplementary Figure 8 — Correlation between YTHDF1 expression and 24 tumor infiltrating lymphocytes (TILs) in KIRP analyzed by the GEPIA database. [file Image_8.tif]

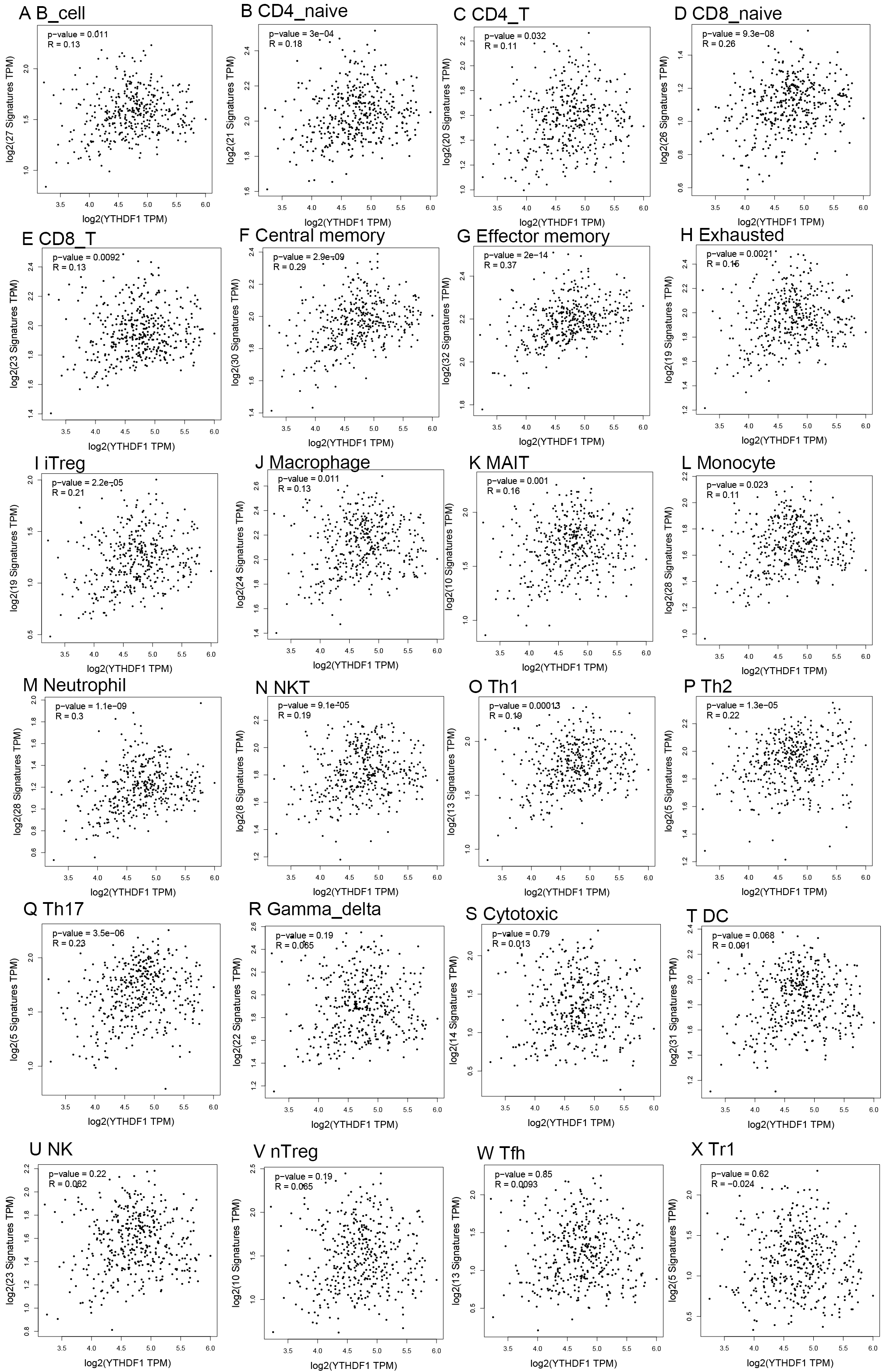

Supplement: Supplementary Figure 9 — Correlation between YTHDF1 expression and 24 tumor infiltrating lymphocytes (TILs) in BLCA analyzed by the GEPIA database. [file Image_9.tif]

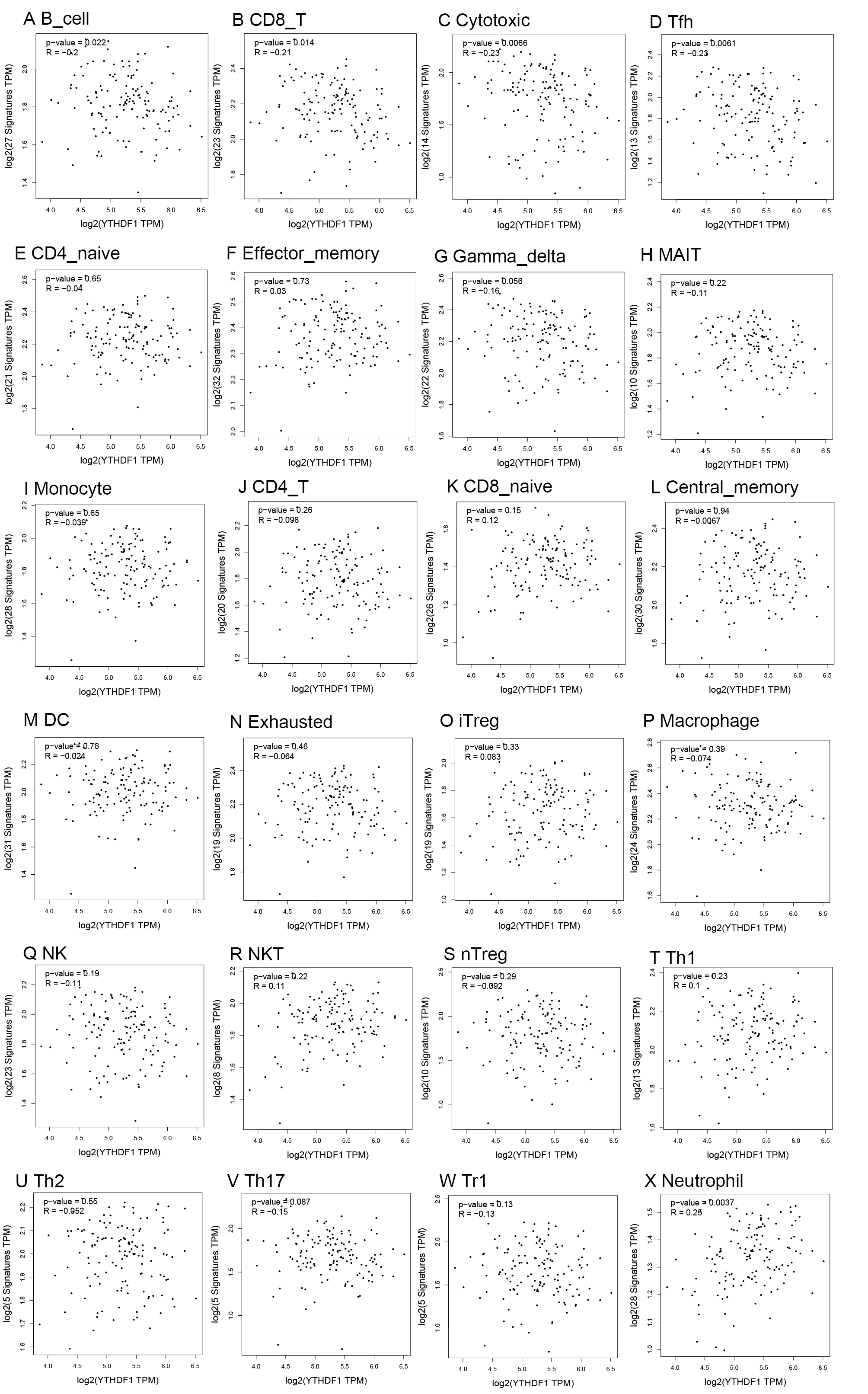

Supplement: Supplementary Figure 10 — Correlation between YTHDF1 expression and 24 tumor infiltrating lymphocytes (TILs) in TGCT analyzed by the GEPIA database. [file Image_10.tif]

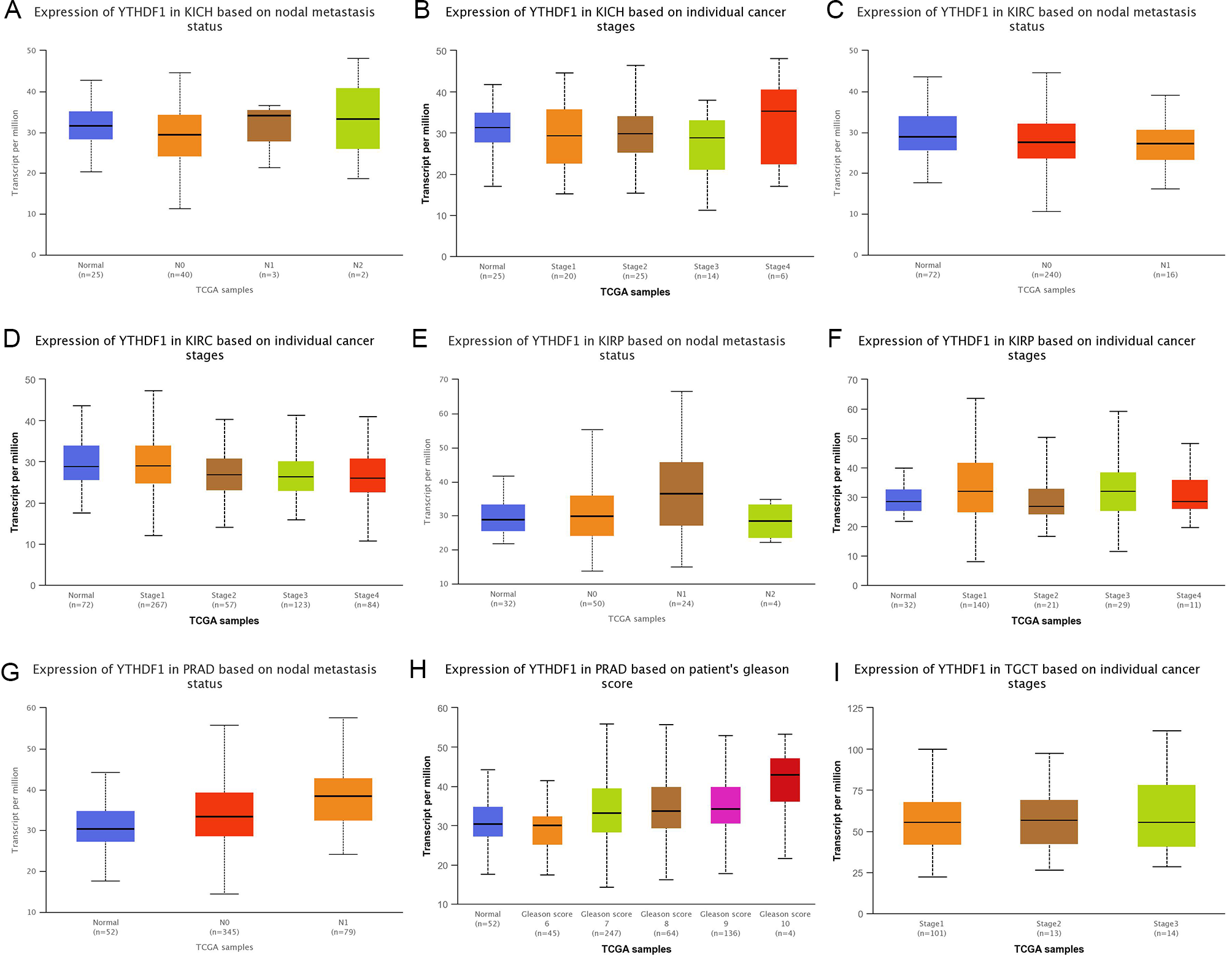

Supplement: Supplementary Figure 11 — YTHDF1 differential expression in five urogenital cancers with different clinical subgroups analyzed by the UALCAN database. [file Image_11.tif]
